# Supplementary material for: Polyphyletic screen defines distinct classes of plant-derived natural products that oppose tauopathy
Source: Life Sci Alliance. 2025 Nov 17;9(2):e202503393. doi: 10.26508/lsa.202503393 (PMC12623141; doi:10.26508/lsa.202503393)
Supplement: Supplementary file 1 [file LSA-2025-03393_TableS1.docx]

**Table S1. List of DEGs in OFA treatment compare to the untreated control in wild-type (N2) worms with the threshold of P≤0.05**

| **GO term** | **Sequence name** | **Public name** | **log2FoldChange** | **pvalue** | **padj** |
| --- | --- | --- | --- | --- | --- |
| **GO:003355** | **Cellular response to stress** | | | | |
|  | F52C12.1 | tdpo-1 | 0.975285927 | 1.31E-05 | 0.000163413 |
|  | F10G8.7 | ercc-1 | 0.719729168 | 2.52E-05 | 0.000287322 |
|  | ZK1128.4 | gtf-2H3 | 0.625802167 | 0.000478206 | 0.003695331 |
|  | F53A3.2 | polh-1 | 0.982447691 | 0.000675968 | 0.004921862 |
|  | Y18H1A.6 | pif-1 | 0.828824144 | 0.001058879 | 0.007032018 |
|  | R10E4.5 | nth-1 | 0.706363284 | 0.001554034 | 0.009539555 |
|  | T02G5.6 | msh-4 pseudogene | 1.720282882 | 0.001630121 | 0.009932225 |
|  | Y73B6BL.14 | Y73B6BL.14 | 1.663103223 | 0.002857711 | 0.015422419 |
|  | Y39A1A.23 | hpr-9 | 0.875824295 | 0.003243532 | 0.017044403 |
|  | R07E5.8 | cku-80 | 0.610194737 | 0.004319584 | 0.021160574 |
|  | Y47G6A.8 | crn-1 | 0.75540936 | 0.007221109 | 0.031153983 |
|  | F57B10.6 | xpg-1 | 0.484520422 | 0.014114072 | 0.050976122 |
| **GO:0006950** | **Response to stress** | | | | |
|  | F49E12.1 | skpo-1 | 1.609065533 | 4.28E-20 | 4.87E-18 |
|  | F22B7.6 | polk-1 | 1.033729244 | 3.55E-08 | 7.70E-07 |
|  | F32A5.2 | skpo-3 | 0.975782034 | 1.07E-05 | 0.000135937 |
|  | F52C12.1 | tdpo-1 | 0.975285927 | 1.31E-05 | 0.000163413 |
|  | K09C8.5 | pxn-2 | 0.962766469 | 1.44E-05 | 0.00017689 |
|  | F10G8.7 | ercc-1 | 0.719729168 | 2.52E-05 | 0.000287322 |
|  | ZK1128.4 | gtf-2H3 | 0.625802167 | 0.000478206 | 0.003695331 |
|  | F53A3.2 | polh-1 | 0.982447691 | 0.000675968 | 0.004921862 |
|  | Y18H1A.6 | pif-1 | 0.828824144 | 0.001058879 | 0.007032018 |
|  | R10E4.5 | nth-1 | 0.706363284 | 0.001554034 | 0.009539555 |
|  | T02G5.6 | msh-4 pseudogene | 1.720282882 | 0.001630121 | 0.009932225 |
|  | Y73B6BL.14 | Y73B6BL.14 | 1.663103223 | 0.002857711 | 0.015422419 |
|  | Y39A1A.23 | hpr-9 | 0.875824295 | 0.003243532 | 0.017044403 |
|  | R07E5.8 | cku-80 | 0.610194737 | 0.004319584 | 0.021160574 |
|  | Y47G6A.8 | crn-1 | 0.75540936 | 0.007221109 | 0.031153983 |
|  | F57B10.6 | xpg-1 | 0.484520422 | 0.014114072 | 0.050976122 |
| **GO:0016579** | **Protein deubiquitination** | | | | |
|  | T22F3.2 | T22F3.2 | 0.634337256 | 3.89E-05 | 0.000419875 |
|  | R10E11.3 | usp-46 | 0.48707005 | 0.000885613 | 0.006083135 |
|  | K02C4.3 | K02C4.3 | 0.816995498 | 0.001279131 | 0.008151227 |
|  | Y106G6H.12 | duo-3 | 0.66678906 | 0.002066202 | 0.011920115 |
|  | F40F12.5 | cyld-1 | 0.373671272 | 0.003076874 | 0.016325661 |
|  | F38B7.5 | duo-1 | 0.928942511 | 0.010208179 | 0.040531021 |
|  | H34C03.2 | usp-4 | 0.587699149 | 0.012322188 | 0.046215253 |
| **GO:0003774** | **Motor activity** | | | | |
|  | K12F2.1 | myo-3 | -0.771986254 | 0.000181435 | 0.001618898 |

| **KEGG term** | **Sequence name** | **Public name** | **log2FoldChange** | **pvalue** | **padj** |
| --- | --- | --- | --- | --- | --- |
| **cel04212** | **Longevity regulating pathway - worm** | | | | |
|  | Y54G11A.6 | ctl-1 | -1.411376138 | 5.26E-10 | 1.54E-08 |
|  | Y54G11A.5 | ctl-2 | -1.130327469 | 6.82E-07 | 1.14E-05 |
|  | F28H6.1 | akt-2 | -0.812475082 | 2.57E-06 | 3.75E-05 |
|  | K04A8.5 | lipl-4 | -1.149378387 | 0.000134722 | 0.001249626 |
|  | VZK822L.1 | fat-6 | -0.710135314 | 0.000229344 | 0.001985712 |
|  | T04C10.4 | atf-4 | -0.791772579 | 0.00023063 | 0.001994738 |
|  | F38E11.2 | hsp-12.6 | -1.33705297 | 0.000264264 | 0.002241864 |
|  | ZK1193.5 | dve-1 | -0.794015713 | 0.000715658 | 0.005153617 |
|  | B0218.3 | pmk-1 | -0.560019911 | 0.000970327 | 0.006528593 |
|  | F52D10.3 | ftt-2 | -0.68777035 | 0.001012776 | 0.006775262 |
|  | W06D12.3 | fat-5 | -0.775146629 | 0.001097502 | 0.007253251 |
|  | F38A6.1 | pha-4 | -1.057097881 | 0.001162445 | 0.007574412 |
|  | K08F4.7 | gst-4 | -0.741361633 | 0.00143617 | 0.008933139 |
|  | F11G11.3 | gst-6 | -0.469747457 | 0.00190617 | 0.011197502 |
|  | F42G8.4 | pmk-3 | -0.562880649 | 0.002266237 | 0.012850635 |
|  | F10D2.9 | fat-7 | -0.948600073 | 0.003974548 | 0.019878805 |
|  | K11G9.6 | mtl-1 | -1.848735551 | 0.005553846 | 0.02562712 |
|  | T07A9.3 | kgb-1 | -0.450551497 | 0.005884599 | 0.026775065 |
|  | C30H6.6 | haf-1 | -0.579903945 | 0.011791723 | 0.044843465 |

| **Down-regulation** | **Gene ID** | **BaseMean** | **log2FoldChange** | **pvalue** | | **padj** |
| --- | --- | --- | --- | --- | --- | --- |
|  | Y47G6A.19 | 98.84031 | -0.75154 | 0.000498 | | 0.009915 |
|  | F15E6.2 | 30.55816 | -1.2605 | 0.000496 | | 0.009895 |
|  | Y54G11A.5 | 46.99531 | -1.17702 | 0.000488 | | 0.009769 |
|  | Y47G6A.15 | 798.5997 | -1.07449 | 0.000483 | | 0.009708 |
|  | C56C10.4 | 17.78229 | -1.66781 | 0.000434 | | 0.008846 |
|  | F32G8.5 | 25.63143 | -1.49567 | 0.000434 | | 0.008846 |
|  | R03G5.1 | 361.8924 | -0.69837 | 0.000432 | | 0.008838 |
|  | C07D10.4 | 61.15635 | -1.0788 | 0.000414 | | 0.008511 |
|  | F32E10.3 | 13.28619 | -2.14287 | 0.0004 | | 0.00828 |
|  | C32D5.12 | 15.83061 | -1.84947 | 0.000401 | | 0.00828 |
|  | Y57A10C.6 | 100.1171 | -1.05156 | 0.000393 | | 0.008146 |
|  | T28H10.3 | 139.6884 | -0.84601 | 0.000387 | | 0.00804 |
|  | D1014.5 | 17.95142 | -1.68613 | 0.000385 | | 0.008035 |
|  | M88.6 | 26.93514 | -1.41058 | 0.000386 | | 0.008035 |
|  | C55B7.4 | 2271.234 | -0.63825 | 0.000373 | | 0.007851 |
|  | C39E9.3 | 44.07998 | -1.80154 | 0.00037 | | 0.007816 |
|  | Y65B4BL.6 | 53.24319 | -1.49837 | 0.000363 | | 0.007669 |
|  | T01D1.3 | 42.64194 | -1.12156 | 0.000359 | | 0.00763 |
|  | F49E12.6 | 21.27601 | -1.6133 | 0.000358 | | 0.007614 |
|  | F01G10.10 | 16.95552 | -1.72922 | 0.000353 | | 0.007529 |
|  | Y51H4A.9 | 64.73392 | -0.94851 | 0.000349 | | 0.007466 |
|  | Y37E3.23 | 10.8967 | -2.87177 | 0.000333 | | 0.007149 |
|  | ZK270.1 | 39.21632 | -1.21775 | 0.000329 | | 0.007078 |
|  | T23G11.6 | 24.48127 | -1.45449 | 0.000315 | | 0.006826 |
|  | F46C8.2 | 30.87283 | -1.50935 | 0.000309 | | 0.006723 |
|  | C47E12.6 | 17.59056 | -1.72242 | 0.000301 | | 0.006567 |
|  | C29F3.2 | 18.63965 | -1.75564 | 0.000298 | | 0.006537 |
|  | H06O01.1 | 529.2364 | -0.50046 | 0.000291 | | 0.006391 |
|  | R11G11.6 | 14.50364 | -1.971 | 0.000287 | | 0.006351 |
|  | T26C5.2 | 13.9228 | -2.34565 | 0.000283 | | 0.006263 |
|  | ZK662.2 | 37.29007 | -1.38644 | 0.000279 | | 0.006211 |
|  | F58G1.5 | 20.40071 | -1.59484 | 0.000259 | | 0.005789 |
|  | Y38A10A.2 | 21.86708 | -1.55094 | 0.000256 | | 0.005723 |
|  | Y62E10A.19 | 22.71134 | -1.51302 | 0.000255 | | 0.005719 |
|  | T07F10.4 | 18.73882 | -1.69657 | 0.000253 | | 0.0057 |
|  | C55F2.1 | 69.34291 | -1.27213 | 0.000248 | | 0.005598 |
|  | ZK662.6 | 33.33431 | -1.46965 | 0.000231 | | 0.005238 |
|  | C26C6.3 | 26.82788 | -1.40146 | 0.000229 | | 0.005219 |
|  | F07H5.5 | 683.1594 | -0.46995 | 0.000222 | | 0.005083 |
|  | T07C4.1 | 84.10405 | -0.90275 | 0.000208 | | 0.004766 |
|  | C05G5.6 | 23.20702 | -1.60954 | 0.000203 | | 0.004663 |
|  | H03E18.1 | 12.32239 | -2.26118 | 0.000199 | | 0.004588 |
|  | C26F1.1 | 52.69218 | -1.1076 | 0.000188 | | 0.004369 |
|  | T10E10.5 | 30.68319 | -1.49992 | 0.000175 | | 0.004122 |
|  | M02D8.4 | 71.8401 | -1.04844 | 0.000175 | | 0.004122 |
|  | C24B5.3 | 20.39958 | -1.65738 | 0.000166 | | 0.003926 |
|  | Y110A2AL.8 | 23.38965 | -1.56961 | 0.000164 | | 0.003901 |
|  | F36A4.10 | 158.5746 | -1.51582 | 0.000164 | | 0.003901 |
|  | F09E10.11 | 107.9402 | -1.10774 | 0.000156 | | 0.003754 |
|  | T10B10.1 | 9.187281 | -3.0642 | 0.000156 | | 0.00375 |
|  | W04G3.1 | 14.90272 | -2.46226 | 0.00015 | | 0.003643 |
|  | T08H10.1 | 191.3132 | -0.79112 | 0.000134 | | 0.003285 |
|  | R07E3.1 | 130.6144 | -0.76182 | 0.000131 | | 0.00324 |
|  | R02E4.3 | 245.4488 | -1.30505 | 0.000125 | | 0.003099 |
|  | ZK909.3 | 182.7738 | -0.70774 | 0.000123 | | 0.003055 |
|  | B0365.9 | 15.32586 | -2.0707 | 0.000116 | | 0.002904 |
|  | F44E2.4 | 12.65997 | -2.30813 | 0.000113 | | 0.002842 |
|  | C29F3.5 | 37.25251 | -1.38443 | 0.000111 | | 0.002796 |
|  | F53H4.3 | 16.80973 | -1.95932 | 0.000106 | | 0.002692 |
|  | ZC101.3 | 16.61229 | -1.93888 | 0.000104 | | 0.002639 |
|  | W05G11.6 | 102.1683 | -0.86691 | 0.000091 | | 0.002337 |
|  | F48E8.1 | 37.47765 | -1.27391 | 9.07E-05 | | 0.002336 |
|  | F44F4.11 | 235.0028 | -0.72551 | 8.27E-05 | | 0.002134 |
|  | C05E7.2 | 141.1792 | -0.76172 | 7.88E-05 | | 0.002051 |
|  | C09E8.2 | 19.65708 | -2 | 7.86E-05 | | 0.00205 |
|  | F28D1.9 | 25.63763 | -1.59334 | 7.24E-05 | | 0.001897 |
|  | K08B12.1 | 14.2921 | -2.27044 | 6.74E-05 | | 0.00178 |
|  | T04G9.5 | 291.1945 | -0.67179 | 6.73E-05 | | 0.00178 |
|  | C54G6.5 | 626.601 | -0.54781 | 6.68E-05 | | 0.001774 |
|  | Y71G12B.18 | 12.70168 | -2.44985 | 6.63E-05 | | 0.001766 |
|  | D1014.6 | 11.04857 | -2.69987 | 6.45E-05 | | 0.001728 |
|  | C30H6.5 | 27.68797 | -1.59718 | 6.36E-05 | | 0.001712 |
|  | C06B3.3 | 48.3069 | -1.18899 | 6.27E-05 | | 0.001693 |
|  | BE0003N10.3 | 12.06019 | -2.66968 | 6.21E-05 | | 0.00168 |
|  | D1037.3 | 405.5145 | -0.5584 | 0.000062 | | 0.00168 |
|  | C15C8.3 | 178.4635 | -0.79284 | 5.89E-05 | | 0.001602 |
|  | BE10.2 | 27.49012 | -1.53854 | 5.57E-05 | | 0.001527 |
|  | D2092.8 | 93.01377 | -1.64006 | 5.53E-05 | | 0.001521 |
|  | F45D3.4 | 92.26855 | -0.96482 | 5.33E-05 | | 0.001481 |
|  | T25G12.5 | 59.87137 | -1.29242 | 5.28E-05 | | 0.001472 |
|  | Y54F10AM.6 | 23.50613 | -1.68946 | 5.24E-05 | | 0.001466 |
|  | C29F9.2 | 50.68158 | -1.47207 | 4.91E-05 | | 0.001384 |
|  | Y37A1B.7 | 22.49876 | -1.84193 | 4.62E-05 | | 0.001313 |
|  | C44H4.3 | 18.55032 | -2.05496 | 4.53E-05 | | 0.00129 |
|  | C06E7.4 | 20.42144 | -1.8559 | 4.49E-05 | | 0.001288 |
|  | F25B5.3 | 271.2009 | -0.68627 | 4.38E-05 | | 0.001259 |
|  | T08G2.3 | 90.81672 | -1.10528 | 4.34E-05 | | 0.00125 |
|  | ZK1290.12 | 24.43301 | -1.93328 | 4.18E-05 | | 0.001218 |
|  | F31C3.1 | 595.0943 | -0.72896 | 4.15E-05 | | 0.001214 |
|  | F26F2.10 | 45.9883 | -1.27962 | 4.13E-05 | | 0.001212 |
|  | F53F4.5 | 72.45538 | -1.02334 | 3.99E-05 | | 0.001175 |
|  | T18H9.1 | 52.395 | -1.2437 | 3.89E-05 | | 0.001148 |
|  | T27A10.6 | 17.72895 | -2.14387 | 3.86E-05 | | 0.001143 |
|  | C15C6.1 | 48.20229 | -1.20699 | 3.64E-05 | | 0.001079 |
|  | F41E6.2 | 686.7673 | -0.84869 | 3.56E-05 | | 0.001058 |
|  | Y53F4B.27 | 19.28431 | -1.966 | 3.49E-05 | | 0.001041 |
|  | F17C8.2 | 34.65953 | -1.61802 | 3.46E-05 | | 0.001038 |
|  | K07E3.3 | 97.35177 | -1.49128 | 3.14E-05 | | 0.000945 |
|  | R74.2 | 31.69218 | -1.56253 | 3.12E-05 | | 0.000941 |
|  | EGAP7.1 | 16.06559 | -2.589 | 2.86E-05 | | 0.000872 |
|  | F30A10.2 | 23.6857 | -1.75949 | 2.84E-05 | | 0.000869 |
|  | T03D8.6 | 39.55895 | -1.47423 | 2.78E-05 | | 0.000853 |
|  | F54C9.1 | 414.0427 | -0.71879 | 2.41E-05 | | 0.000744 |
|  | T01D1.4 | 89.61474 | -0.93535 | 2.26E-05 | | 0.000703 |
|  | F09B12.1 | 17.74498 | -2.14533 | 0.000022 | | 0.000689 |
|  | T10E10.1 | 69.98686 | -1.19501 | 2.14E-05 | | 0.000674 |
|  | D2085.1 | 36.39335 | -1.43546 | 2.13E-05 | | 0.000672 |
|  | F52D1.3 | 18.96665 | -2.09245 | 0.00002 | | 0.000641 |
|  | T06D8.10 | 19.11248 | -2.02694 | 1.98E-05 | | 0.000637 |
|  | F13D11.4 | 29.59917 | -1.59416 | 1.81E-05 | | 0.000583 |
|  | Y75B8A.28 | 26.4143 | -1.90426 | 1.74E-05 | | 0.000565 |
|  | R12E2.15 | 473.5592 | -1.28897 | 1.65E-05 | | 0.000538 |
|  | T06A4.3 | 26.01888 | -1.72042 | 0.000016 | | 0.000529 |
|  | F47B10.7 | 103.9005 | -1.24838 | 1.34E-05 | | 0.000449 |
|  | R07B1.13 | 33.50489 | -1.74542 | 1.33E-05 | | 0.000447 |
|  | R12E2.14 | 529.0086 | -1.219 | 1.21E-05 | | 0.000408 |
|  | C24B9.9 | 27.7636 | -1.74476 | 1.18E-05 | | 0.000401 |
|  | R09B5.8 | 22.0502 | -1.93061 | 1.16E-05 | | 0.000396 |
|  | T28F4.5 | 539.6688 | -0.9901 | 1.11E-05 | | 0.00038 |
|  | C28H8.11 | 123.7778 | -0.96624 | 0.000011 | | 0.00038 |
|  | W04G3.2 | 15.41186 | -2.77693 | 1.09E-05 | | 0.000379 |
|  | C01B10.11 | 69.50606 | -1.11771 | 1.05E-05 | | 0.000365 |
|  | T19A5.3 | 21.24561 | -1.99677 | 9.83E-06 | | 0.000345 |
|  | M03A8.1 | 187.1123 | -0.96022 | 9.36E-06 | | 0.00033 |
|  | F44A6.5 | 83.06313 | -1.19787 | 9.28E-06 | | 0.000328 |
|  | F26B1.4 | 43.59376 | -1.37786 | 9.04E-06 | | 0.000321 |
|  | C54F6.14 | 23.42159 | -1.97512 | 8.65E-06 | | 0.00031 |
|  | F26A10.28 | 17.0902 | -2.57652 | 8.18E-06 | | 0.000296 |
|  | F09B12.3 | 77.13451 | -1.05658 | 8.05E-06 | | 0.000293 |
|  | W06B11.4 | 32.6429 | -1.69669 | 7.73E-06 | | 0.000282 |
|  | Y97E10C.1 | 130.6287 | -0.90337 | 7.52E-06 | | 0.000277 |
|  | F32A11.6 | 29.51117 | -1.76351 | 7.1E-06 | | 0.000262 |
|  | T23F2.1 | 21.53737 | -2.02237 | 6.92E-06 | | 0.000257 |
|  | ZC434.3 | 23.3722 | -1.91066 | 6.7E-06 | | 0.00025 |
|  | T14B4.6 | 20.64429 | -2.58812 | 6.43E-06 | | 0.000241 |
|  | C45B2.7 | 24.12111 | -2.09328 | 6.4E-06 | | 0.00024 |
|  | C56A3.2 | 229.7193 | -0.85755 | 5.74E-06 | | 0.00022 |
|  | W05H9.3 | 188.6631 | -0.90053 | 5.51E-06 | | 0.000213 |
|  | F09G8.6 | 257.4692 | -1.00903 | 5.36E-06 | | 0.000207 |
|  | R12C12.10 | 36.77512 | -1.69475 | 4.7E-06 | | 0.000185 |
|  | C14F11.6 | 30.71207 | -1.79466 | 4.67E-06 | | 0.000184 |
|  | F42G9.2 | 83.80526 | -1.07308 | 4.57E-06 | | 0.000181 |
|  | T03G6.1 | 25.3507 | -1.88474 | 3.85E-06 | | 0.000153 |
|  | F08F1.6 | 44.9164 | -1.74573 | 3.6E-06 | | 0.000144 |
|  | T02C5.1 | 206.8398 | -0.94967 | 3.57E-06 | | 0.000143 |
|  | C02D5.1 | 30.41612 | -1.73221 | 3.54E-06 | | 0.000143 |
|  | R05H5.8 | 64.75851 | -1.33962 | 3.45E-06 | | 0.00014 |
|  | F46E10.9 | 136.419 | -1.10149 | 3.26E-06 | | 0.000132 |
|  | ZK84.1 | 193.2023 | -1.78827 | 2.98E-06 | | 0.000122 |
|  | C34D4.15 | 69.39288 | -1.33996 | 2.88E-06 | | 0.000119 |
|  | Y38F1A.3 | 26.65821 | -1.8692 | 2.79E-06 | | 0.000115 |
|  | C06G1.1 | 36.2274 | -1.63108 | 2.52E-06 | | 0.000105 |
|  | F10F2.3 | 36.34339 | -1.60299 | 2.38E-06 | | 9.95E-05 |
|  | F26G1.5 | 111.6079 | -1.06943 | 1.93E-06 | | 8.12E-05 |
|  | T01B10.2 | 336.6909 | -0.90806 | 1.63E-06 | | 6.95E-05 |
|  | T05C12.10 | 22.50357 | -2.24243 | 1.47E-06 | | 6.39E-05 |
|  | C05E7.1 | 69.23107 | -1.38634 | 1.47E-06 | | 6.39E-05 |
|  | C09E8.3 | 47.99666 | -2.21439 | 1.26E-06 | | 5.55E-05 |
|  | F56D3.1 | 34.00726 | -1.77517 | 1.25E-06 | | 0.000055 |
|  | C31H2.2 | 20.52363 | -2.48631 | 1.21E-06 | | 5.35E-05 |
|  | C33G3.3 | 51.75962 | -1.56103 | 1.09E-06 | | 4.83E-05 |
|  | T21D12.2 | 25.90198 | -2.48526 | 1.07E-06 | | 4.77E-05 |
|  | F53B6.9 | 54.84676 | -1.79301 | 1.03E-06 | | 4.66E-05 |
|  | D1086.3 | 47.74563 | -1.47887 | 1.03E-06 | | 4.66E-05 |
|  | ZK377.1 | 37.97178 | -1.90001 | 1.02E-06 | | 4.63E-05 |
|  | Y11D7A.5 | 53.01559 | -1.78015 | 9.64E-07 | | 4.41E-05 |
|  | F32A5.4 | 67.67045 | -1.34185 | 8.75E-07 | | 4.02E-05 |
|  | ZK154.1 | 49.29212 | -1.49221 | 8.63E-07 | | 3.98E-05 |
|  | R12E2.7 | 731.2946 | -1.29091 | 7.93E-07 | | 3.68E-05 |
|  | C34E7.4 | 129.724 | -1.05867 | 7.94E-07 | | 3.68E-05 |
|  | F09F9.2 | 29.09806 | -1.92833 | 7.61E-07 | | 3.57E-05 |
|  | C29F7.2 | 62.25268 | -1.35248 | 7.63E-07 | | 3.57E-05 |
|  | T17H7.7 | 36.3037 | -1.89385 | 6.99E-07 | | 3.33E-05 |
|  | C50F2.6 | 48.78658 | -1.5478 | 6.97E-07 | | 3.33E-05 |
|  | ZC328.1 | 30.90961 | -1.89631 | 5.88E-07 | | 2.84E-05 |
|  | ZC13.4 | 35.85283 | -1.72053 | 5.57E-07 | | 0.000027 |
|  | F46C8.6 | 27.048 | -2.55841 | 5.06E-07 | | 2.47E-05 |
|  | Y47D7A.13 | 485.7287 | -1.33566 | 4.03E-07 | | 1.99E-05 |
|  | T10E9.3 | 92.23823 | -1.30923 | 3.89E-07 | | 1.94E-05 |
|  | Y71G12B.25 | 29.9504 | -1.93189 | 3.66E-07 | | 1.83E-05 |
|  | F58H1.2 | 38.26812 | -1.91455 | 3.49E-07 | | 1.76E-05 |
|  | T10E10.6 | 38.47777 | -1.71151 | 3.46E-07 | | 1.76E-05 |
|  | F52D1.1 | 36.64257 | -1.79875 | 3.32E-07 | | 1.69E-05 |
|  | VC5.3 | 286.6646 | -1.05871 | 3.27E-07 | | 1.68E-05 |
|  | H04M03.4 | 36.95539 | -1.85196 | 3.19E-07 | | 1.65E-05 |
|  | T10E10.2 | 192.5456 | -0.95091 | 3.14E-07 | | 1.64E-05 |
|  | F08G5.4 | 358.7222 | -1.47422 | 2.9E-07 | | 1.52E-05 |
|  | C34G6.6 | 22.50482 | -2.47295 | 2.76E-07 | | 1.45E-05 |
|  | C14A4.9 | 34.73294 | -1.81347 | 2.59E-07 | | 1.37E-05 |
|  | F49C5.11 | 48.2341 | -1.55019 | 2.24E-07 | | 0.000012 |
|  | R09A8.4 | 30.93947 | -1.9908 | 2.18E-07 | | 1.17E-05 |
|  | C09G5.6 | 29.3823 | -2.26127 | 1.85E-07 | | 1.01E-05 |
|  | K01D12.9 | 81.27869 | -1.24969 | 1.81E-07 | | 9.94E-06 |
|  | F33A8.7 | 50.05643 | -1.54601 | 1.71E-07 | | 9.48E-06 |
|  | W04G3.3 | 25.25519 | -2.74872 | 1.28E-07 | | 7.17E-06 |
|  | Y65B4BL.1 | 28.3864 | -2.44049 | 1.26E-07 | | 7.13E-06 |
|  | ZK1025.3 | 36.31772 | -1.89628 | 1.24E-07 | | 0.000007 |
|  | C42D8.5 | 30.40339 | -2.10735 | 1.1E-07 | | 6.27E-06 |
|  | T28C6.6 | 64.23484 | -1.45231 | 1.1E-07 | | 6.27E-06 |
|  | H42K12.3 | 27.92316 | -2.28886 | 1.08E-07 | | 6.2E-06 |
|  | C02F5.14 | 77.1234 | -1.83768 | 1E-07 | | 5.83E-06 |
|  | F47F6.1 | 32.93098 | -1.96574 | 9.82E-08 | | 5.76E-06 |
|  | F55C10.3 | 244.1307 | -0.88463 | 9.55E-08 | | 5.63E-06 |
|  | F46C8.8 | 34.08077 | -1.94159 | 9.23E-08 | | 5.48E-06 |
|  | T06G6.6 | 34.94081 | -1.94484 | 9.15E-08 | | 5.46E-06 |
|  | C26B9.3 | 67.89701 | -1.38276 | 7.54E-08 | | 4.56E-06 |
|  | W04G3.8 | 22.97504 | -2.86048 | 5.5E-08 | | 3.46E-06 |
|  | C52G5.2 | 122.2121 | -1.24985 | 5.53E-08 | | 3.46E-06 |
|  | R11A5.7 | 65.69504 | -1.51511 | 4.86E-08 | | 3.08E-06 |
|  | F52B11.3 | 26.59856 | -2.53006 | 4.21E-08 | | 2.74E-06 |
|  | C15C7.5 | 189.2066 | -1.2626 | 2.74E-08 | | 1.82E-06 |
|  | F41C6.5 | 37.87124 | -1.93345 | 2.13E-08 | | 1.43E-06 |
|  | F58E6.13 | 89.11195 | -1.68687 | 1.99E-08 | | 1.36E-06 |
|  | Y48G8AL.12 | 1038.036 | -1.4006 | 1.94E-08 | | 1.33E-06 |
|  | C34H4.4 | 678.7094 | -1.65549 | 1.64E-08 | | 1.13E-06 |
|  | ZK1307.1 | 111.9473 | -1.24412 | 1.05E-08 | | 7.36E-07 |
|  | C04G6.2 | 90.2112 | -1.59553 | 9.69E-09 | | 6.93E-07 |
|  | C07A12.4 | 505.5063 | -0.87644 | 9.18E-09 | | 6.68E-07 |
|  | ZK1290.8 | 38.54816 | -2.42487 | 8.2E-09 | | 6.12E-07 |
|  | M153.1 | 87.87819 | -1.55889 | 7.93E-09 | | 5.96E-07 |
|  | C02E7.6 | 228.1742 | -1.70002 | 6.06E-09 | | 4.66E-07 |
|  | Y75B8A.20 | 57.83296 | -1.72691 | 5.95E-09 | | 4.61E-07 |
|  | T28C6.4 | 90.29598 | -1.48717 | 3.82E-09 | | 3.05E-07 |
|  | F13H8.5 | 52.58065 | -1.84282 | 3.73E-09 | | 3E-07 |
|  | F33D4.6 | 39.57002 | -2.29243 | 3.64E-09 | | 2.96E-07 |
|  | C03G6.15 | 67.30735 | -1.52563 | 3.57E-09 | | 2.92E-07 |
|  | R07E5.4 | 78.02569 | -1.49195 | 3.32E-09 | | 2.76E-07 |
|  | C50B6.4 | 847.1104 | -1.24157 | 3.15E-09 | | 2.64E-07 |
|  | F38A3.2 | 769.62 | -1.52083 | 2.27E-09 | | 1.92E-07 |
|  | Y47D3B.10 | 67.43111 | -1.58496 | 1.74E-09 | | 1.48E-07 |
|  | F57B7.3 | 58.01623 | -2.02271 | 1.47E-09 | | 1.28E-07 |
|  | H10E21.4 | 260.4482 | -1.32884 | 1.2E-09 | | 1.05E-07 |
|  | F29C12.1 | 37.82939 | -2.17118 | 8.79E-10 | | 7.89E-08 |
|  | ZK617.2 | 165.5473 | -1.24573 | 6.69E-10 | | 6.12E-08 |
|  | Y66A7A.6 | 76.73847 | -1.62202 | 6.22E-10 | | 5.74E-08 |
|  | C38C6.6 | 130.0381 | -1.6992 | 5.57E-10 | | 5.2E-08 |
|  | F57B9.9 | 47.39064 | -1.997 | 3.95E-10 | | 3.78E-08 |
|  | Y11D7A.11 | 346.7764 | -1.21089 | 3.59E-10 | | 3.47E-08 |
|  | C49F8.3 | 156.4924 | -1.17858 | 2.51E-10 | | 2.47E-08 |
|  | Y47D7A.5 | 82.70207 | -2.03738 | 1.87E-10 | | 1.88E-08 |
|  | T12D8.5 | 81.94239 | -1.74498 | 1.68E-10 | | 1.7E-08 |
|  | F22D6.10 | 334.3994 | -1.34473 | 1.16E-10 | | 1.2E-08 |
|  | F49H12.5 | 115.6516 | -1.50635 | 9.81E-11 | | 1.03E-08 |
|  | C31C9.2 | 90.00565 | -1.49499 | 6.55E-11 | | 7.07E-09 |
|  | C29E4.1 | 60.67467 | -1.86173 | 6.38E-11 | | 6.96E-09 |
|  | Y47D7A.15 | 748.1908 | -1.22701 | 5.99E-11 | | 6.68E-09 |
|  | T02E9.2 | 51.59854 | -2.11941 | 5.66E-11 | | 6.38E-09 |
|  | C05E4.9 | 284.861 | -1.41318 | 4.93E-11 | | 5.75E-09 |
|  | W05G11.3 | 404.3634 | -1.47623 | 3.22E-11 | | 3.79E-09 |
|  | B0222.8 | 227.7585 | -1.33964 | 2.19E-11 | | 2.61E-09 |
|  | C25B8.3 | 697.7449 | -1.17564 | 1.98E-11 | | 2.39E-09 |
|  | C44C10.1 | 180.427 | -1.28306 | 1.82E-11 | | 2.23E-09 |
|  | F10D11.6 | 92.71309 | -1.62016 | 1.37E-11 | | 1.72E-09 |
|  | T19C3.2 | 133.5622 | -1.66099 | 1.26E-11 | | 1.63E-09 |
|  | C09G5.3 | 76.75919 | -1.75778 | 9.58E-12 | | 1.26E-09 |
|  | Y11D7A.9 | 80.37082 | -1.70892 | 7.65E-12 | | 1.02E-09 |
|  | F53B1.4 | 60.11715 | -2.19115 | 7.16E-12 | | 9.69E-10 |
|  | Y2H9A.3 | 615.7694 | -1.39934 | 5.94E-12 | | 8.15E-10 |
|  | F57H12.6 | 111.2097 | -1.92005 | 5.31E-12 | | 7.38E-10 |
|  | F19C7.7 | 82.27216 | -1.86898 | 4.79E-12 | | 6.76E-10 |
|  | ZC373.6 | 147.1849 | -1.60029 | 4.47E-12 | | 6.39E-10 |
|  | C02E7.7 | 276.9426 | -1.53512 | 4.27E-12 | | 6.19E-10 |
|  | T03E6.7 | 772.4248 | -1.02344 | 3.96E-12 | | 5.82E-10 |
|  | B0222.7 | 247.625 | -1.32277 | 2.75E-12 | | 4.1E-10 |
|  | T22H6.2 | 67.9839 | -1.89514 | 9.15E-13 | | 1.43E-10 |
|  | ZK180.5 | 287.4679 | -1.89039 | 8.26E-13 | | 1.31E-10 |
|  | ZK836.1 | 152.5451 | -1.71218 | 7E-13 | | 1.13E-10 |
|  | ZK678.5 | 87.08961 | -1.77948 | 5.91E-13 | | 9.68E-11 |
|  | B0222.6 | 446.3288 | -1.38724 | 4.42E-13 | | 7.48E-11 |
|  | K08C9.4 | 163.2493 | -1.5719 | 4.33E-13 | | 7.44E-11 |
|  | F55C10.2 | 279.7095 | -1.54529 | 4.06E-13 | | 7.14E-11 |
|  | F15B9.8 | 142.9135 | -1.4834 | 3.54E-13 | | 6.41E-11 |
|  | F29B9.9 | 114.359 | -1.7161 | 2.65E-13 | | 5.07E-11 |
|  | K02E11.10 | 93.56154 | -1.8225 | 1.13E-13 | | 2.25E-11 |
|  | W08F4.6 | 123.0337 | -1.88703 | 8.32E-14 | | 1.69E-11 |
|  | Y102A11A.5 | 124.6767 | -1.86538 | 6.58E-14 | | 1.36E-11 |
|  | Y65B4BR.6 | 379.2726 | -1.83568 | 5.11E-14 | | 1.09E-11 |
|  | C29F4.1 | 912.5444 | -1.56656 | 5.17E-14 | | 1.09E-11 |
|  | F32B5.8 | 430.2156 | -1.52706 | 1.94E-14 | | 4.48E-12 |
|  | F35G2.4 | 139.0968 | -1.61658 | 1.22E-14 | | 2.88E-12 |
|  | C35B8.1 | 756.8363 | -1.73317 | 3.83E-15 | | 9.25E-13 |
|  | F53F1.4 | 1166.674 | -1.54058 | 6.45E-16 | | 1.64E-13 |
|  | K09H9.3 | 474.1967 | -1.71591 | 5.36E-16 | | 1.39E-13 |
|  | C05C8.3 | 170.5358 | -1.52992 | 4.46E-16 | | 1.19E-13 |
|  | Y59A8B.20 | 157.4632 | -1.55717 | 4.07E-16 | | 1.12E-13 |
|  | C46A5.3 | 561.4377 | -1.67928 | 3.03E-16 | | 8.79E-14 |
|  | M195.1 | 870.6277 | -1.75887 | 1.15E-16 | | 3.53E-14 |
|  | T11F9.9 | 129.4435 | -1.95654 | 6.19E-17 | | 1.96E-14 |
|  | F58F6.1 | 494.7357 | -1.63501 | 4.09E-17 | | 1.34E-14 |
|  | F53F1.5 | 278.4702 | -1.9279 | 1.01E-17 | | 3.42E-15 |
|  | T01B7.7 | 118.0473 | -1.97838 | 8.29E-18 | | 2.93E-15 |
|  | F41F3.3 | 1950.623 | -1.71044 | 8.37E-18 | | 2.93E-15 |
|  | ZK180.6 | 97.79678 | -2.08428 | 5.54E-18 | | 2.08E-15 |
|  | H23N18.5 | 333.1125 | -1.60662 | 5.61E-19 | | 2.28E-16 |
|  | M03A1.7 | 151.5993 | -2.30422 | 2.45E-20 | | 1.04E-17 |
|  | F11G11.10 | 176.9741 | -2.18448 | 1.07E-21 | | 4.72E-19 |
|  | Y73B6BL.34 | 696.2559 | -1.92189 | 7.71E-22 | | 3.56E-19 |
|  | ZK265.2 | 472.0619 | -1.88917 | 7.06E-23 | | 3.41E-20 |
|  | Y38C1BA.3 | 190.2984 | -1.97321 | 3.23E-23 | | 1.64E-20 |
|  | F59E12.12 | 192.8165 | -1.813 | 1.66E-23 | | 8.86E-21 |
|  | B0491.2 | 198.0617 | -2.0684 | 6.88E-24 | | 3.88E-21 |
|  | Y57A10A.23 | 551.1085 | -1.59115 | 4.13E-24 | | 2.47E-21 |
|  | ZK1290.3 | 150.8768 | -2.13446 | 2.53E-24 | | 1.71E-21 |
|  | T19B10.2 | 231.7493 | -1.89481 | 1.88E-24 | | 1.36E-21 |
|  | C01B12.1 | 190.8432 | -2.12051 | 5.03E-28 | | 3.93E-25 |
|  | Y57A10A.11 | 350.7894 | -2.04079 | 3.09E-28 | | 2.61E-25 |
|  | E01G4.6 | 184.3922 | -2.16004 | 1.62E-28 | | 1.5E-25 |
|  | Y54E10BL.2 | 215.1719 | -2.06189 | 5.43E-30 | | 5.51E-27 |
|  | Y49F6B.10 | 523.7423 | -2.02504 | 1.14E-30 | | 1.29E-27 |
|  | C52D10.13 | 700.7219 | -2.06635 | 1.01E-30 | | 1.28E-27 |
|  | ZK1010.7 | 453.6698 | -1.90827 | 9.55E-31 | | 1.28E-27 |
|  | F54C9.4 | 661.4627 | -2.02723 | 2.23E-34 | | 3.77E-31 |
|  | F27C1.8 | 571.0721 | -2.16228 | 1.1E-35 | | 3.43E-32 |
|  | Y41E3.2 | 605.4345 | -2.11915 | 1.47E-35 | | 3.43E-32 |
|  | F11G11.12 | 998.5213 | -1.98953 | 1.69E-35 | | 3.43E-32 |
|  | F30B5.1 | 540.1639 | -2.11175 | 4.92E-37 | | 2.5E-33 |
|  | F23H12.4 | 365.5352 | -2.10595 | 1.49E-42 | | 1.51E-38 |
| **Up-regulation** | **Gene ID** | **BaseMean** | **log2FoldChange** | | **pvalue** | **padj** |
|  | T28H11.1 | 189.5711 | 0.887261 | | 0.000491 | 0.009807 |
|  | T07A9.6 | 36.18995 | 1.320809 | | 0.000488 | 0.009769 |
|  | C38D4.6 | 34.58222 | 1.191194 | | 0.000482 | 0.009708 |
|  | T21C9.13 | 19.9955 | 1.54421 | | 0.000476 | 0.009602 |
|  | Y18D10A.20 | 181.2249 | 0.664236 | | 0.000446 | 0.009028 |
|  | R04D3.3 | 9.50404 | 2.434746 | | 0.000447 | 0.009028 |
|  | C48E7.1 | 107.9471 | 0.73469 | | 0.000443 | 0.008993 |
|  | ZK228.4 | 69.65963 | 1.193053 | | 0.000438 | 0.00891 |
|  | C03C10.3 | 102.9464 | 0.833473 | | 0.00043 | 0.008816 |
|  | F32D1.7 | 13.91497 | 1.993003 | | 0.000413 | 0.008497 |
|  | ZC168.5 | 88.01545 | 0.864535 | | 0.000402 | 0.008295 |
|  | T25B9.1 | 23.62721 | 1.468187 | | 0.00038 | 0.007941 |
|  | C35D10.11 | 395.8689 | 0.49533 | | 0.000377 | 0.007905 |
|  | T25G3.2 | 21.2887 | 1.607695 | | 0.000377 | 0.007905 |
|  | B0273.2 | 17.02928 | 1.809201 | | 0.000363 | 0.007669 |
|  | C14F11.4 | 72.46617 | 0.909048 | | 0.000353 | 0.007529 |
|  | K07H8.6 | 937.6495 | 0.897697 | | 0.000332 | 0.007135 |
|  | C14F11.5 | 34.62688 | 1.285584 | | 0.000329 | 0.007078 |
|  | EEED8.3 | 30.1822 | 1.294631 | | 0.000323 | 0.006988 |
|  | T11F9.3 | 42.18702 | 1.186024 | | 0.000303 | 0.006601 |
|  | C05D9.9 | 25.4379 | 1.51074 | | 0.000299 | 0.006544 |
|  | ZK354.4 | 136.341 | 0.903538 | | 0.00029 | 0.006388 |
|  | T22D1.5 | 52.09174 | 1.160251 | | 0.000288 | 0.006353 |
|  | C50H2.10 | 41.56178 | 1.485712 | | 0.000281 | 0.006234 |
|  | K10C2.8 | 25.44616 | 1.607663 | | 0.000264 | 0.005892 |
|  | C05A9.1 | 24.45953 | 1.63566 | | 0.000251 | 0.005656 |
|  | H01M10.3 | 12.13183 | 2.218959 | | 0.000241 | 0.005459 |
|  | C49F5.7 | 128.6597 | 1.589522 | | 0.000231 | 0.005238 |
|  | W06B4.1 | 27.68637 | 1.454831 | | 0.000205 | 0.004696 |
|  | F35C11.5 | 32.76843 | 1.355072 | | 0.000194 | 0.004489 |
|  | C29F7.3 | 129.3703 | 0.745233 | | 0.000189 | 0.004387 |
|  | F58A6.8 | 259.9399 | 0.815748 | | 0.000188 | 0.004365 |
|  | ZK354.1 | 96.52794 | 0.941936 | | 0.000184 | 0.004297 |
|  | C17E4.3 | 27.86025 | 1.422071 | | 0.000183 | 0.004276 |
|  | Y43F8C.2 | 48.78115 | 1.547494 | | 0.000179 | 0.004185 |
|  | F56F3.1 | 113.0098 | 1.119509 | | 0.000173 | 0.004076 |
|  | ZK228.3 | 30.52355 | 1.392167 | | 0.000165 | 0.003913 |
|  | Y105C5A.8 | 13.944 | 2.208757 | | 0.000159 | 0.003786 |
|  | C14B1.9 | 54.55691 | 1.17286 | | 0.000158 | 0.003776 |
|  | DY3.8 | 30.98867 | 1.349861 | | 0.000154 | 0.00372 |
|  | T12G3.6 | 27.39094 | 1.428334 | | 0.000151 | 0.003656 |
|  | F57B10.12 | 38.6504 | 1.389582 | | 0.000151 | 0.00365 |
|  | R05F9.8 | 356.1433 | 0.813268 | | 0.000149 | 0.003627 |
|  | F57A8.8 | 25.76977 | 1.838653 | | 0.000146 | 0.003568 |
|  | ZK546.6 | 321.5761 | 0.799368 | | 0.00014 | 0.003412 |
|  | Y48A6B.7 | 59.78227 | 1.325367 | | 0.000139 | 0.003409 |
|  | F26F12.1 | 1197.998 | 0.965668 | | 0.000132 | 0.00324 |
|  | C35B1.4 | 54.94647 | 1.061615 | | 0.000132 | 0.00324 |
|  | F38A5.5 | 22.98124 | 1.799715 | | 0.00013 | 0.003225 |
|  | W05E7.1 | 42.03676 | 1.423397 | | 0.000116 | 0.002905 |
|  | B0213.2 | 46.15418 | 1.741959 | | 0.000112 | 0.002818 |
|  | AH6.5 | 51.89503 | 1.467868 | | 0.000111 | 0.002796 |
|  | Y22D7AR.10 | 12.30543 | 2.684221 | | 0.00011 | 0.002796 |
|  | C07H6.5 | 364.4836 | 1.231718 | | 9.96E-05 | 0.002547 |
|  | F14F7.1 | 50.68374 | 1.139193 | | 9.93E-05 | 0.002545 |
|  | Y43E12A.1 | 48.00797 | 1.236414 | | 8.24E-05 | 0.002132 |
|  | T21E8.1 | 29.68956 | 1.499746 | | 8.02E-05 | 0.002082 |
|  | R01H2.3 | 29.14833 | 1.600278 | | 7.29E-05 | 0.001907 |
|  | T27C10.7 | 107.9003 | 0.877983 | | 7.17E-05 | 0.001884 |
|  | C39B5.5 | 248.6651 | 0.808613 | | 6.80E-05 | 0.001791 |
|  | C01G8.1 | 30.13263 | 1.578271 | | 6.51E-05 | 0.001737 |
|  | T23G11.2 | 36.37476 | 1.39233 | | 6.43E-05 | 0.001726 |
|  | F13G11.3 | 75.32014 | 1.00296 | | 5.82E-05 | 0.001588 |
|  | ZK354.5 | 171.7743 | 0.927413 | | 5.74E-05 | 0.00157 |
|  | C10C5.3 | 57.26392 | 1.089467 | | 5.51E-05 | 0.001519 |
|  | F53G12.5 | 47.4949 | 1.386611 | | 5.50E-05 | 0.001519 |
|  | F43C11.3 | 120.9159 | 1.648422 | | 5.46E-05 | 0.001513 |
|  | C06A6.4 | 169.4757 | 0.875002 | | 5.02E-05 | 0.001411 |
|  | C14B1.2 | 52.61409 | 1.177891 | | 5.03E-05 | 0.001411 |
|  | H02I12.5 | 25.53799 | 1.783513 | | 4.86E-05 | 0.001374 |
|  | F23H12.9 | 37.41182 | 1.377993 | | 4.83E-05 | 0.001368 |
|  | W02F12.3 | 20.63835 | 1.866651 | | 4.51E-05 | 0.00129 |
|  | C09G9.6 | 52.37987 | 1.581265 | | 4.33E-05 | 0.00125 |
|  | T09F3.3 | 65.21387 | 1.170852 | | 4.28E-05 | 0.001241 |
|  | K08F4.8 | 101.935 | 1.006863 | | 4.21E-05 | 0.001223 |
|  | C09B9.6 | 124.5633 | 0.961629 | | 3.48E-05 | 0.001041 |
|  | ZC412.7 | 74.58966 | 1.799437 | | 3.03E-05 | 0.000918 |
|  | ZK858.3 | 43.41702 | 1.360575 | | 2.94E-05 | 0.000894 |
|  | C50H2.12 | 49.6021 | 1.581794 | | 2.78E-05 | 0.000853 |
|  | T07C4.5 | 801.2346 | 0.882599 | | 2.62E-05 | 0.000809 |
|  | Y18D10A.17 | 271.6316 | 1.044906 | | 2.32E-05 | 0.000719 |
|  | Y45F10C.3 | 51.29512 | 1.256446 | | 2.21E-05 | 0.000689 |
|  | ZC404.8 | 91.35337 | 1.480064 | | 2.17E-05 | 0.000681 |
|  | M01E11.5 | 443.7311 | 0.962729 | | 2.08E-05 | 0.000663 |
|  | T05F1.2 | 43.3961 | 1.52888 | | 2.09E-05 | 0.000663 |
|  | C25A8.4 | 24.08941 | 1.778872 | | 2.08E-05 | 0.000662 |
|  | T05A10.5 | 30.04408 | 1.915982 | | 1.77E-05 | 0.000571 |
|  | T06E4.4 | 562.6544 | 1.251678 | | 1.72E-05 | 0.000561 |
|  | T08A9.11 | 56.62691 | 1.220341 | | 1.65E-05 | 0.000538 |
|  | F38A3.1 | 1403.399 | 1.030825 | | 1.63E-05 | 0.000536 |
|  | Y45F10A.2 | 59.25891 | 1.634962 | | 1.61E-05 | 0.000531 |
|  | F59D8.2 | 50.52432 | 1.242999 | | 1.45E-05 | 0.000482 |
|  | H13N06.6 | 26.89021 | 1.71853 | | 1.44E-05 | 0.00048 |
|  | F38A5.14 | 29.87224 | 1.813255 | | 1.44E-05 | 0.000479 |
|  | Y41C4A.19 | 33.13053 | 1.554703 | | 1.19E-05 | 0.000401 |
|  | F27C8.6 | 72.54165 | 1.42363 | | 1.14E-05 | 0.000388 |
|  | T04G9.7 | 60.87834 | 1.21626 | | 1.10E-05 | 0.000379 |
|  | ZK1193.1 | 927.078 | 1.039921 | | 1.08E-05 | 0.000374 |
|  | F48E3.4 | 42.64876 | 1.373663 | | 1.04E-05 | 0.000364 |
|  | Y73B6BL.38 | 57.79663 | 1.542182 | | 8.89E-06 | 0.000316 |
|  | H31G24.4 | 64.5347 | 1.362321 | | 8.75E-06 | 0.000313 |
|  | F17E9.4 | 96.72409 | 1.394301 | | 8.65E-06 | 0.00031 |
|  | C05C10.5 | 34.38743 | 1.709993 | | 8.61E-06 | 0.00031 |
|  | F42A8.1 | 31.78033 | 1.718584 | | 7.63E-06 | 0.00028 |
|  | Y51F10.2 | 33.39875 | 1.773885 | | 6.93E-06 | 0.000257 |
|  | F14H3.6 | 31.12732 | 1.679918 | | 6.36E-06 | 0.00024 |
|  | W03C9.7 | 32.09373 | 1.781987 | | 6.24E-06 | 0.000236 |
|  | Y87G2A.5 | 148.2951 | 0.872024 | | 6.08E-06 | 0.000231 |
|  | F49E12.1 | 34.2489 | 1.585333 | | 5.82E-06 | 0.000222 |
|  | ZK813.2 | 34.30352 | 2.446681 | | 5.80E-06 | 0.000222 |
|  | F59A6.12 | 19.84474 | 2.41002 | | 5.13E-06 | 0.0002 |
|  | C24F3.6 | 1622.417 | 1.0873 | | 5.01E-06 | 0.000195 |
|  | W02A2.7 | 77.92363 | 1.597421 | | 4.74E-06 | 0.000186 |
|  | B0213.3 | 37.09757 | 2.198597 | | 3.09E-06 | 0.000126 |
|  | T13F3.6 | 280.3472 | 1.537676 | | 2.95E-06 | 0.000121 |
|  | F26D10.10 | 38.48872 | 1.669297 | | 2.37E-06 | 9.95E-05 |
|  | F57C2.4 | 36.90036 | 1.651465 | | 1.85E-06 | 7.84E-05 |
|  | C45B2.1 | 406.4616 | 1.716375 | | 1.84E-06 | 7.83E-05 |
|  | F56B3.1 | 55.28588 | 1.488033 | | 1.63E-06 | 6.95E-05 |
|  | C42D8.2 | 845.984 | 1.112203 | | 1.62E-06 | 6.94E-05 |
|  | F55B11.3 | 29.20392 | 2.174654 | | 1.50E-06 | 6.48E-05 |
|  | T06E6.2 | 93.17354 | 1.503591 | | 1.46E-06 | 6.38E-05 |
|  | F22B3.4 | 76.78369 | 1.685386 | | 1.05E-06 | 4.73E-05 |
|  | B0513.4 | 36.40457 | 1.698192 | | 7.33E-07 | 3.46E-05 |
|  | T05G5.7 | 56.53177 | 1.430062 | | 7.21E-07 | 3.42E-05 |
|  | T11F8.3 | 51.89769 | 1.761302 | | 6.13E-07 | 2.95E-05 |
|  | W03F11.1 | 18.0305 | 3.01546 | | 4.28E-07 | 2.10E-05 |
|  | K10B2.3 | 59.3192 | 1.442006 | | 3.94E-07 | 1.95E-05 |
|  | F18A1.7 | 73.40626 | 1.378211 | | 3.61E-07 | 1.81E-05 |
|  | K11D12.13 | 19.83095 | 2.601663 | | 3.22E-07 | 1.66E-05 |
|  | W02D9.7 | 40.84203 | 2.49945 | | 2.42E-07 | 1.28E-05 |
|  | T25E12.5 | 52.0765 | 1.714865 | | 2.35E-07 | 1.26E-05 |
|  | ZK783.2 | 81.49249 | 1.228103 | | 2.13E-07 | 1.16E-05 |
|  | D1086.11 | 129.8026 | 1.110398 | | 1.81E-07 | 9.94E-06 |
|  | Y75B12B.1 | 78.03121 | 1.2835 | | 1.39E-07 | 7.75E-06 |
|  | K07A1.6 | 41.67287 | 2.161009 | | 1.05E-07 | 6.08E-06 |
|  | Y105C5B.5 | 71.05723 | 1.438035 | | 9.09E-08 | 5.46E-06 |
|  | C28C12.2 | 73.86444 | 1.608648 | | 7.11E-08 | 4.32E-06 |
|  | F54C9.8 | 60.42834 | 1.734086 | | 6.60E-08 | 4.04E-06 |
|  | Y69H2.14 | 77.74591 | 1.43851 | | 6.31E-08 | 3.88E-06 |
|  | T21C9.3 | 55.62267 | 1.801421 | | 5.70E-08 | 3.53E-06 |
|  | D1086.6 | 40.34462 | 1.994204 | | 5.59E-08 | 3.48E-06 |
|  | C50B6.2 | 98.09262 | 1.628318 | | 4.54E-08 | 2.90E-06 |
|  | C28D4.3 | 66.81827 | 1.655011 | | 4.53E-08 | 2.90E-06 |
|  | Y62H9A.4 | 53.82155 | 2.056458 | | 4.49E-08 | 2.90E-06 |
|  | T01C3.3 | 67.36137 | 1.461801 | | 3.75E-08 | 2.46E-06 |
|  | K09F5.2 | 59.01745 | 1.511792 | | 2.93E-08 | 1.93E-06 |
|  | W05F2.3 | 54.21935 | 1.73926 | | 2.22E-08 | 1.48E-06 |
|  | B0244.8 | 66.81212 | 1.77428 | | 2.04E-08 | 1.38E-06 |
|  | C10G8.4 | 26.55431 | 2.813312 | | 1.49E-08 | 1.04E-06 |
|  | H02I12.1 | 62.05199 | 1.896403 | | 1.03E-08 | 7.28E-07 |
|  | C39D10.7 | 56.3769 | 1.620996 | | 1.01E-08 | 7.20E-07 |
|  | B0024.1 | 109.781 | 1.364159 | | 9.41E-09 | 6.77E-07 |
|  | K02G10.7 | 56.93095 | 2.02151 | | 9.08E-09 | 6.68E-07 |
|  | Y62H9A.3 | 41.00451 | 2.293082 | | 9.21E-09 | 6.68E-07 |
|  | ZC513.6 | 50.76552 | 1.907551 | | 8.79E-09 | 6.51E-07 |
|  | Y45F10C.4 | 208.1721 | 0.994866 | | 6.55E-09 | 4.96E-07 |
|  | C09G5.5 | 705.0769 | 1.948833 | | 6.37E-09 | 4.86E-07 |
|  | F16B4.4 | 314.6461 | 1.163384 | | 5.34E-09 | 4.17E-07 |
|  | T04F8.8 | 222.7457 | 1.673512 | | 4.54E-09 | 3.57E-07 |
|  | B0280.5 | 229.5375 | 1.813438 | | 4.10E-09 | 3.25E-07 |
|  | ZC373.2 | 122.874 | 2.272338 | | 3.38E-09 | 2.79E-07 |
|  | Y57G11B.5 | 68.72198 | 1.680527 | | 1.58E-09 | 1.36E-07 |
|  | Y37D8A.19 | 124.5147 | 2.309151 | | 1.13E-09 | 9.93E-08 |
|  | K02D7.3 | 26.37953 | 2.977707 | | 1.07E-09 | 9.57E-08 |
|  | K03H1.4 | 275.2736 | 0.952814 | | 8.23E-10 | 7.46E-08 |
|  | C15A11.6 | 374.4099 | 1.06348 | | 5.59E-10 | 5.20E-08 |
|  | F52E1.1 | 114.9657 | 1.708043 | | 4.25E-10 | 4.03E-08 |
|  | D1054.11 | 149.1209 | 2.230115 | | 2.63E-10 | 2.56E-08 |
|  | ZK813.7 | 93.16317 | 2.316654 | | 1.88E-10 | 1.88E-08 |
|  | C25A1.8 | 134.2881 | 1.793883 | | 1.41E-10 | 1.44E-08 |
|  | Y62H9A.6 | 120.9047 | 2.205629 | | 7.47E-11 | 7.90E-09 |
|  | F11H8.3 | 207.9774 | 2.492402 | | 7.01E-11 | 7.49E-09 |
|  | R06C7.4 | 113.042 | 1.594648 | | 6.30E-11 | 6.95E-09 |
|  | C44B7.5 | 97.06079 | 1.472763 | | 5.31E-11 | 6.05E-09 |
|  | Y47G6A.33 | 116.9744 | 1.661884 | | 5.21E-11 | 6.01E-09 |
|  | C34F6.2 | 786.7839 | 1.420748 | | 1.59E-11 | 1.96E-09 |
|  | C07G2.1 | 83.36238 | 1.868604 | | 1.28E-11 | 1.63E-09 |
|  | ZK813.1 | 54.43893 | 2.657307 | | 1.27E-11 | 1.63E-09 |
|  | C15A11.5 | 363.8504 | 1.25697 | | 2.45E-12 | 3.71E-10 |
|  | F55B11.2 | 122.3621 | 1.609239 | | 1.43E-12 | 2.19E-10 |
|  | T15B7.3 | 36.20665 | 3.664837 | | 5.15E-13 | 8.57E-11 |
|  | F11G11.11 | 858.0475 | 1.521009 | | 4.08E-13 | 7.14E-11 |
|  | ZK813.3 | 78.53597 | 2.555073 | | 3.08E-13 | 5.69E-11 |
|  | F43D9.4 | 259.2587 | 1.816864 | | 3.01E-13 | 5.66E-11 |
|  | C34F6.3 | 237.413 | 1.699782 | | 2.52E-13 | 4.93E-11 |
|  | T15B7.4 | 99.20356 | 2.321351 | | 4.58E-14 | 1.01E-11 |
|  | Y62H9A.5 | 148.0884 | 2.210524 | | 2.85E-14 | 6.42E-12 |
|  | D1054.10 | 254.2484 | 1.727159 | | 1.03E-15 | 2.56E-13 |
|  | C53B4.5 | 589.5944 | 2.20639 | | 4.01E-16 | 1.12E-13 |
|  | C44B12.5 | 204.0139 | 1.590729 | | 1.57E-16 | 4.70E-14 |
|  | C44B12.1 | 200.3423 | 1.552962 | | 9.50E-19 | 3.71E-16 |
|  | C04F6.1 | 222.4981 | 1.918829 | | 2.80E-24 | 1.78E-21 |

**List of DEGs in OFB treatment compare to the untreated control in wild-type (N2) worms with the threshold of P≤0.05**

| **GO term** | **Sequence name** | **Public name** | **log2FoldChange** | **pvalue** | **padj** |
| --- | --- | --- | --- | --- | --- |
| **GO:0033554** | **Cellular response to stress** | | | | |
|  | F52C12.1 | tdpo-1 | 0.923499667 | 1.21E-05 | 0.000129242 |
|  | F10G8.7 | ercc-1 | 0.648530656 | 0.000146129 | 0.001215012 |
|  | ZK1128.4 | gtf-2H3 | 0.560758803 | 0.001941426 | 0.011281461 |
|  | F53A3.2 | polh-1 | 0.918726682 | 0.001530666 | 0.009249914 |
|  | R10E4.5 | nth-1 | 0.658715783 | 0.002133803 | 0.012234387 |
|  | Y73B6BL.14 | Y73B6BL.14 | 1.633811929 | 0.002900107 | 0.015702912 |
|  | ZK20.3 | rad-23 | 0.429896368 | 0.011153833 | 0.046766462 |
| **GO:0006950** | **Response to stress** | | | |  |
|  | F49E12.1 | skpo-1 | 1.683031928 | 5.44E-23 | 4.35E-21 |
|  | F22B7.6 | polk-1 | 0.938206593 | 4.05E-07 | 5.91E-06 |
|  | F32A5.2 | skpo-3 | 1.416723932 | 1.34E-10 | 3.27E-09 |
|  | F52C12.1 | tdpo-1 | 0.923499667 | 1.21E-05 | 0.000129242 |
|  | K09C8.5 | pxn-2 | 1.717007604 | 1.18E-17 | 5.72E-16 |
|  | F10G8.7 | ercc-1 | 0.648530656 | 0.000146129 | 0.001215012 |
|  | ZK1128.4 | gtf-2H3 | 0.560758803 | 0.001941426 | 0.011281461 |
|  | F53A3.2 | polh-1 | 0.918726682 | 0.001530666 | 0.009249914 |
|  | ZK20.3 | rad-23 | 0.429896368 | 0.011153833 | 0.046766462 |
|  | Y74C9A.5 | sesn-1 | 0.458474772 | 0.002936703 | 0.015872578 |
| **GO:0003774** | **Motor activity** | | | | |
|  | K12F2.1 | myo-3 | -0.792777835 | 5.91611E-05 | 0.000545252 |

| **KEGG term** | **Sequence name** | **Public name** | **log2FoldChange** | **pvalue** | **padj** |
| --- | --- | --- | --- | --- | --- |
| **cel04212** | **Longevity regulating pathway - worm** | | | | |
|  | Y54G11A.6 | ctl-1 | -0.876239776 | 0.00074773 | 0.005048585 |
|  | Y54G11A.5 | ctl-2 | -0.854095522 | 0.000156282 | 0.001291125 |
|  | F28H6.1 | akt-2 | -0.961574454 | 1.56E-08 | 2.83E-07 |
|  | K04A8.5 | lipl-4 | -1.443157465 | 2.51E-06 | 3.09E-05 |
|  | VZK822L.1 | fat-6 | -0.799849244 | 4.93E-05 | 0.000463118 |
|  | T04C10.4 | atf-4 | -0.602592235 | 0.004839519 | 0.023891163 |
|  | F38E11.2 | hsp-12.6 | -1.068291499 | 0.002652937 | 0.014569423 |
|  | ZK1193.5 | dve-1 | -0.59747121 | 0.010200472 | 0.043495583 |
|  | B0218.3 | pmk-1 | -0.569729823 | 0.001633358 | 0.009764007 |
|  | W06D12.3 | fat-5 | -1.16405304 | 1.71E-06 | 2.19E-05 |
|  | K08F4.7 | gst-4 | -0.601523801 | 0.011092326 | 0.046552791 |
|  | F11G11.3 | gst-6 | -0.544191819 | 0.000515531 | 0.003673866 |
|  | F42G8.4 | pmk-3 | -0.556631963 | 0.002669489 | 0.014642554 |
|  | K11G9.6 | mtl-1 | -2.111293714 | 0.000326035 | 0.002472377 |
|  | C30H6.6 | haf-1 | -0.715617468 | 0.002927595 | 0.015828073 |

| **Down-regulation** | | **Gene ID** | | **BaseMean** | **log2FoldChange** | **pvalue** | **padj** |
| --- | --- | --- | --- | --- | --- | --- | --- |
|  | | C52D10.25 | | 4.31214499 | -5.5733905 | 0.00039796 | 0.007181 |
|  | | C24B9.9 | | 22.6269054 | -4.2256506 | 1.92E-10 | 1.36E-08 |
|  | | T10B10.1 | | 8.74656893 | -4.068535 | 8.09E-05 | 0.00181403 |
|  | | W04G3.2 | | 14.5182381 | -3.7818503 | 1.94E-06 | 6.35E-05 |
|  | | T14B4.6 | | 19.1330025 | -3.7635897 | 3.85E-07 | 1.43E-05 |
|  | | W04G3.3 | | 23.7597232 | -3.7534568 | 7.12E-09 | 4.10E-07 |
|  | | W04G3.1 | | 13.6871879 | -3.6904462 | 1.22E-05 | 0.0003381 |
|  | | F46C8.6 | | 25.0751508 | -3.6892502 | 1.25E-08 | 6.64E-07 |
|  | | W04G3.8 | | 21.949209 | -3.6302691 | 7.97E-09 | 4.43E-07 |
|  | | T14B4.7 | | 8.57536098 | -3.593988 | 0.00030851 | 0.00587392 |
|  | | T21D12.2 | | 24.0851598 | -3.4901617 | 3.02E-08 | 1.51E-06 |
|  | | Y65B4BM.2 | | 7.92544252 | -3.4697802 | 0.0005205 | 0.0090874 |
|  | | EGAP7.1 | | 15.1763679 | -3.4014111 | 5.33E-06 | 0.00015811 |
|  | | K08B12.1 | | 13.0432547 | -3.3728228 | 3.84E-06 | 0.00011987 |
|  | | Y75B7AR.1 | | 14.3334701 | -3.311126 | 0.00036387 | 0.00669427 |
|  | | ZK1290.8 | | 36.1325846 | -3.2465239 | 7.17E-11 | 5.40E-09 |
|  | | C01F1.5 | | 8.24802524 | -3.1794897 | 0.00046291 | 0.00822048 |
|  | | T26C5.2 | | 13.0266433 | -3.1584082 | 4.93E-05 | 0.00116733 |
|  | | Y46G5A.36 | | 7.93653654 | -3.1168309 | 0.00029883 | 0.00572135 |
|  | | F23H12.5 | | 7.921207 | -3.1144968 | 0.00038934 | 0.0070821 |
|  | | Y51H7C.13 | | 104.115993 | -3.1071828 | 2.41E-06 | 7.73E-05 |
|  | | C09E8.3 | | 44.5191908 | -3.0634792 | 7.66E-09 | 4.28E-07 |
|  | | Y37A1B.7 | | 19.8085833 | -3.0514034 | 7.19E-08 | 3.22E-06 |
|  | | C31H2.2 | | 19.6389791 | -3.0375447 | 1.56E-07 | 6.35E-06 |
|  | | F33D4.6 | | 37.2964277 | -2.9534988 | 1.97E-11 | 1.59E-09 |
|  | | Y65B4BL.1 | | 27.2226315 | -2.9516692 | 1.07E-08 | 5.79E-07 |
|  | | W01F3.3 | | 8.58604936 | -2.9494745 | 0.00020653 | 0.00413639 |
|  | | F52B11.3 | | 25.7441292 | -2.9486911 | 5.57E-09 | 3.26E-07 |
|  | | C09G5.6 | | 27.7218916 | -2.8974332 | 6.33E-09 | 3.66E-07 |
|  | | M03A1.7 | | 143.918866 | -2.8790199 | 1.13E-26 | 3.37E-24 |
|  | | ZC123.1 | | 8.08670373 | -2.8516219 | 0.00052677 | 0.0091263 |
|  | | C54F6.14 | | 21.3276611 | -2.8328019 | 4.79E-08 | 2.26E-06 |
|  | | F35G2.6 | | 25.2716808 | -2.8308545 | 0.00011323 | 0.00244084 |
|  | | T05C12.10 | | 21.291577 | -2.8305536 | 7.02E-08 | 3.16E-06 |
|  | | H03E18.1 | | 11.7186577 | -2.7949164 | 4.34E-05 | 0.00105425 |
|  | | B0365.9 | | 14.2127221 | -2.7814954 | 4.48E-06 | 0.00013801 |
|  | | C34G6.6 | | 21.9511987 | -2.7808623 | 5.98E-08 | 2.75E-06 |
|  | | Y102A11A.5 | | 112.257086 | -2.7792252 | 1.34E-24 | 3.37E-22 |
|  | | F32G8.5 | | 21.791282 | -2.7687875 | 5.81E-08 | 2.69E-06 |
|  | | F33D11.3 | | 10.2422161 | -2.7663486 | 8.44E-05 | 0.00188069 |
|  | | T06G6.6 | | 31.8980148 | -2.7606844 | 3.06E-11 | 2.40E-09 |
|  | | ZK617.2 | | 134.422991 | -2.7352684 | 4.03E-36 | 2.28E-33 |
|  | | F32E10.3 | | 12.5365778 | -2.7320971 | 7.82E-05 | 0.00176289 |
|  | | F30A10.2 | | 21.1389542 | -2.7182445 | 3.44E-08 | 1.67E-06 |
|  | | Y47D7A.5 | | 77.0433712 | -2.7171937 | 3.28E-14 | 3.46E-12 |
|  | | F29C12.1 | | 35.8320188 | -2.707656 | 2.19E-12 | 1.93E-10 |
|  | | D1014.6 | | 11.072635 | -2.7001796 | 7.35E-05 | 0.00167501 |
|  | | F57H12.3 | | 13.5255234 | -2.69997 | 7.77E-05 | 0.00175859 |
|  | | F57H12.6 | | 101.689863 | -2.6932505 | 2.23E-19 | 3.99E-17 |
|  | | H42K12.3 | | 26.9002321 | -2.6906002 | 9.99E-09 | 5.48E-07 |
|  | | F22F4.1 | | 9.73395841 | -2.6825014 | 0.0003413 | 0.00639349 |
|  | | F26A10.28 | | 17.0005561 | -2.6786278 | 1.22E-05 | 0.0003381 |
|  | | F11G11.10 | | 168.525167 | -2.6761881 | 4.60E-30 | 1.67E-27 |
|  | | F58H1.2 | | 35.0480222 | -2.6704866 | 1.86E-10 | 1.33E-08 |
|  | | F09B12.1 | | 16.8356554 | -2.6627137 | 1.92E-06 | 6.31E-05 |
|  | | T27A10.6 | | 16.8294604 | -2.6622024 | 4.72E-06 | 0.00014366 |
|  | | C45B2.7 | | 22.7656626 | -2.6567778 | 3.92E-07 | 1.44E-05 |
|  | | ZC434.3 | | 21.4726326 | -2.6490242 | 3.90E-08 | 1.86E-06 |
|  | | ZK1290.12 | | 22.5955159 | -2.6441351 | 1.46E-06 | 4.88E-05 |
|  | | T02E9.2 | | 49.0223953 | -2.6126436 | 4.57E-14 | 4.68E-12 |
|  | | F35G2.4 | | 122.679291 | -2.5982979 | 1.83E-31 | 7.64E-29 |
|  | | B0213.15 | | 9.25189113 | -2.5968523 | 0.00031402 | 0.00595168 |
|  | | F53B1.4 | | 57.7575363 | -2.5952865 | 2.98E-14 | 3.21E-12 |
|  | | C04F1.1 | | 10.252348 | -2.5709906 | 0.00029492 | 0.00567984 |
|  | | Y75B8A.20 | | 52.1567531 | -2.5638822 | 2.28E-14 | 2.52E-12 |
|  | | C14A4.9 | | 31.7151081 | -2.5618224 | 1.08E-10 | 7.89E-09 |
|  | | R09E10.13 | | 18.0257298 | -2.5527951 | 1.91E-05 | 0.00049257 |
|  | | F44E2.4 | | 12.3814514 | -2.5514031 | 4.58E-05 | 0.00109847 |
|  | | T03G6.1 | | 23.4544314 | -2.5381291 | 2.26E-08 | 1.15E-06 |
|  | | F57B9.9 | | 44.5870058 | -2.5349138 | 3.91E-14 | 4.09E-12 |
|  | | ZK1290.3 | | 144.652928 | -2.5319175 | 1.51E-35 | 8.13E-33 |
|  | | ZC328.1 | | 28.7262644 | -2.5200362 | 2.17E-09 | 1.37E-07 |
|  | | F09F9.2 | | 27.2369975 | -2.4964772 | 1.11E-08 | 5.96E-07 |
|  | | E01G4.6 | | 178.150498 | -2.4911474 | 6.94E-37 | 4.36E-34 |
|  | | T23F2.1 | | 20.4699315 | -2.4772334 | 3.48E-07 | 1.31E-05 |
|  | | T22D1.18 | | 14.8877084 | -2.4540567 | 0.00014578 | 0.00304391 |
|  | | Y71G12B.18 | | 12.7252329 | -2.449787 | 0.00012639 | 0.00268865 |
|  | | D2024.4 | | 10.5668987 | -2.4440933 | 0.00022098 | 0.00440241 |
|  | | R11G11.6 | | 13.714276 | -2.4409303 | 4.72E-05 | 0.00112862 |
|  | | C42D8.5 | | 29.3777529 | -2.4321869 | 6.06E-09 | 3.53E-07 |
|  | | C06G4.6 | | 16.6865246 | -2.4213538 | 6.26E-06 | 0.00018201 |
|  | | R09B5.8 | | 20.8060036 | -2.4175569 | 5.81E-07 | 2.07E-05 |
|  | | B0491.2 | | 190.80166 | -2.4119196 | 1.47E-31 | 6.37E-29 |
|  | | ZK1025.3 | | 34.1647724 | -2.4092053 | 7.68E-10 | 5.07E-08 |
|  | | F13B12.4 | | 11.381992 | -2.4086268 | 0.00030423 | 0.00581493 |
|  | | T01B7.7 | | 112.415123 | -2.4025693 | 5.95E-24 | 1.40E-21 |
|  | | F28C6.5 | | 10.2332311 | -2.3892688 | 0.00039347 | 0.00712659 |
|  | | F09E10.11 | | 87.9403391 | -2.3876961 | 8.46E-13 | 7.65E-11 |
|  | | C14F11.6 | | 28.5457141 | -2.3829896 | 3.39E-08 | 1.65E-06 |
|  | | F53B6.9 | | 50.7755205 | -2.3755894 | 7.57E-09 | 4.27E-07 |
|  | | Y38C1BA.3 | | 181.605514 | -2.3743126 | 1.73E-36 | 1.03E-33 |
|  | | F57B7.3 | | 55.7839486 | -2.3739584 | 8.71E-14 | 8.71E-12 |
|  | | W08F4.6 | | 116.169344 | -2.366995 | 1.31E-19 | 2.38E-17 |
|  | | F23H12.4 | | 355.361161 | -2.3657412 | 9.07E-68 | 1.28E-64 |
|  | | C01B12.1 | | 186.073663 | -2.3593513 | 5.33E-39 | 3.54E-36 |
|  | | C32D5.12 | | 14.8739227 | -2.3344341 | 4.40E-05 | 0.00106248 |
|  | | ZC250.3 | | 9.90762207 | -2.3334809 | 0.00057061 | 0.00981069 |
|  | | ZC101.3 | | 15.8492813 | -2.3330965 | 1.24E-05 | 0.00034113 |
|  | | F27C1.8 | | 561.463702 | -2.3293295 | 4.90E-79 | 1.38E-75 |
|  | | F08F1.6 | | 41.5063406 | -2.3285854 | 3.14E-08 | 1.56E-06 |
|  | | F47F6.1 | | 31.5456943 | -2.3244807 | 1.31E-09 | 8.45E-08 |
|  | | Y11D7A.5 | | 49.2794162 | -2.3239886 | 1.46E-08 | 7.69E-07 |
|  | | C02F5.14 | | 72.4276422 | -2.3120645 | 1.84E-10 | 1.33E-08 |
|  | | F52D1.3 | | 18.5087306 | -2.3036582 | 5.63E-06 | 0.00016548 |
|  | | Y53F4B.27 | | 18.5048186 | -2.3033769 | 3.94E-06 | 0.000122 |
|  | | Y57A10A.11 | | 341.046523 | -2.2946936 | 9.73E-49 | 9.16E-46 |
|  | | F58E6.13 | | 81.9991192 | -2.2868663 | 3.37E-12 | 2.92E-10 |
|  | | C47E12.6 | | 16.3415801 | -2.2810374 | 1.53E-05 | 0.00040264 |
|  | | F25H8.6 | | 10.5633319 | -2.2794678 | 0.00043259 | 0.0077442 |
|  | | K02E11.10 | | 88.3127934 | -2.2786456 | 1.01E-19 | 1.87E-17 |
|  | | ZK662.6 | | 29.5956565 | -2.2685522 | 6.79E-07 | 2.40E-05 |
|  | | R10H1.5 | | 12.3855109 | -2.2653042 | 0.0001314 | 0.00278478 |
|  | | F49D11.6 | | 16.1953479 | -2.2649616 | 3.49E-05 | 0.00085923 |
|  | | Y49F6B.10 | | 509.360183 | -2.2641778 | 1.32E-70 | 2.49E-67 |
|  | | Y54E10BL.2 | | 210.383268 | -2.2639854 | 1.20E-42 | 9.00E-40 |
|  | | C52D10.13 | | 685.927737 | -2.2604522 | 1.33E-80 | 7.54E-77 |
|  | | H04M03.4 | | 35.1445355 | -2.2603913 | 7.45E-09 | 4.25E-07 |
|  | | C05G5.7 | | 11.3967897 | -2.2597572 | 0.00028487 | 0.00551009 |
|  | | Y54F10AM.6 | | 21.7875942 | -2.2556254 | 8.48E-07 | 2.96E-05 |
|  | | R07B1.13 | | 31.2456301 | -2.2540986 | 2.26E-07 | 8.91E-06 |
|  | | Y41E3.2 | | 597.208996 | -2.251022 | 2.96E-79 | 1.12E-75 |
|  | | C26C6.3 | | 23.6167313 | -2.2506154 | 2.57E-07 | 9.97E-06 |
|  | | ZK180.6 | | 96.1101494 | -2.2460477 | 2.16E-22 | 4.69E-20 |
|  | | ZK265.2 | | 453.410312 | -2.2180374 | 1.92E-41 | 1.35E-38 |
|  | | D1014.5 | | 16.6834166 | -2.2170792 | 1.70E-05 | 0.0004416 |
|  | | F30B5.1 | | 534.967862 | -2.2087098 | 1.26E-67 | 1.58E-64 |
|  | | W06B11.4 | | 30.4043591 | -2.2065013 | 7.44E-08 | 3.31E-06 |
|  | | F59E12.12 | | 183.257987 | -2.2060346 | 6.46E-34 | 3.04E-31 |
|  | | F54C9.4 | | 649.7081 | -2.1898671 | 4.83E-76 | 1.09E-72 |
|  | | T12D8.5 | | 77.1679804 | -2.1805708 | 2.12E-14 | 2.37E-12 |
|  | | ZK836.1 | | 143.364544 | -2.1801655 | 2.82E-16 | 4.14E-14 |
|  | | R12C12.10 | | 34.3720752 | -2.1742028 | 8.80E-08 | 3.84E-06 |
|  | | F27E5.4 | | 17.1560601 | -2.1717853 | 6.92E-05 | 0.00158242 |
|  | | C35B8.1 | | 714.347516 | -2.1694687 | 6.89E-28 | 2.29E-25 |
|  | | F49C5.11 | | 44.0947 | -2.1652701 | 1.22E-11 | 1.02E-09 |
|  | | F11G11.12 | | 978.442242 | -2.16416 | 6.57E-89 | 7.42E-85 |
|  | | F49E12.12 | | 18.8448166 | -2.1599242 | 3.52E-05 | 0.00086167 |
|  | | F32D1.11 | | 12.5405774 | -2.1578585 | 0.00041741 | 0.00748418 |
|  | | ZK1010.7 | | 439.882325 | -2.1574759 | 6.27E-69 | 1.01E-65 |
|  | | Y74E4A.1 | | 12.5379631 | -2.1571465 | 0.00036134 | 0.00666952 |
|  | | ZC13.4 | | 33.8399213 | -2.1469969 | 2.70E-09 | 1.67E-07 |
|  | | Y11D7A.9 | | 75.6453625 | -2.1456229 | 1.11E-16 | 1.69E-14 |
|  | | Y38F1A.3 | | 25.7559805 | -2.1420945 | 1.70E-07 | 6.80E-06 |
|  | | Y73B6BL.34 | | 677.979784 | -2.131397 | 8.99E-43 | 7.26E-40 |
|  | | C44H4.3 | | 18.4739929 | -2.1256529 | 3.75E-05 | 0.00091594 |
|  | | ZC373.6 | | 136.306941 | -2.1245082 | 1.12E-16 | 1.69E-14 |
|  | | T03D8.6 | | 35.9607809 | -2.1201495 | 1.05E-07 | 4.49E-06 |
|  | | Y38A10A.2 | | 20.1373271 | -2.116874 | 5.06E-06 | 0.00015289 |
|  | | C02D5.1 | | 28.8836046 | -2.1163387 | 6.76E-08 | 3.05E-06 |
|  | | F19C7.7 | | 79.6359258 | -2.1157423 | 2.52E-14 | 2.74E-12 |
|  | | C32H11.5 | | 19.1599079 | -2.1072358 | 1.45E-05 | 0.00038709 |
|  | | ZK678.5 | | 83.3898026 | -2.1026333 | 2.52E-18 | 4.25E-16 |
|  | | T06D8.10 | | 18.9940094 | -2.0916646 | 1.01E-05 | 0.0002841 |
|  | | C06E7.4 | | 19.8176946 | -2.0881276 | 6.26E-06 | 0.00018201 |
|  | | BE0003N10.3 | | 12.8925079 | -2.0833878 | 0.0005801 | 0.00992856 |
|  | | H23N18.5 | | 311.03409 | -2.0733555 | 1.08E-30 | 4.22E-28 |
|  | | F01G10.10 | | 16.1819123 | -2.0674775 | 4.40E-05 | 0.00106248 |
|  | | T07F10.4 | | 17.8229739 | -2.0615737 | 2.43E-05 | 0.00061777 |
|  | | K09H9.3 | | 452.757503 | -2.0596492 | 1.61E-24 | 3.95E-22 |
|  | | T19A5.3 | | 21.1371566 | -2.0543869 | 3.87E-06 | 0.00012036 |
|  | | C46A5.3 | | 533.185273 | -2.0478704 | 8.58E-48 | 7.45E-45 |
|  | | F46C8.8 | | 33.6832867 | -2.047235 | 9.69E-09 | 5.34E-07 |
|  | | T11F9.9 | | 128.574324 | -2.0320352 | 1.59E-23 | 3.59E-21 |
|  | | F53H4.3 | | 16.6826893 | -2.0299736 | 6.56E-05 | 0.00151456 |
|  | | F41C6.5 | | 37.4829349 | -2.0276158 | 2.64E-09 | 1.64E-07 |
|  | | F58F6.1 | | 467.579792 | -2.0256564 | 8.00E-63 | 9.04E-60 |
|  | | F26F2.10 | | 40.7533204 | -2.025282 | 7.60E-09 | 4.27E-07 |
|  | | Y18H1A.9 | | 13.2066267 | -2.011513 | 0.00032063 | 0.0060565 |
|  | | F56D3.1 | | 32.9981486 | -2.0105817 | 1.41E-07 | 5.88E-06 |
|  | | Y59A8B.20 | | 147.426173 | -1.9974378 | 8.17E-26 | 2.25E-23 |
|  | | R09A8.4 | | 31.0347506 | -1.9924406 | 1.63E-07 | 6.58E-06 |
|  | | C49H3.16 | | 13.8797267 | -1.9911892 | 0.00039569 | 0.00715147 |
|  | | F29B9.9 | | 110.006728 | -1.9880375 | 5.62E-18 | 9.06E-16 |
|  | | C24B5.3 | | 19.4785093 | -1.981006 | 1.59E-05 | 0.00041857 |
|  | | C09G5.3 | | 74.4807994 | -1.9762773 | 1.28E-14 | 1.49E-12 |
|  | | C26F1.5 | | 16.1771505 | -1.974799 | 7.89E-05 | 0.00177611 |
|  | | Y57A10A.23 | | 520.392693 | -1.9730189 | 3.62E-31 | 1.46E-28 |
|  | | Y71G12B.25 | | 29.8793478 | -1.9718959 | 1.54E-07 | 6.28E-06 |
|  | | F58G1.5 | | 19.3136919 | -1.9656981 | 2.40E-05 | 0.00061093 |
|  | | F53F1.5 | | 277.414211 | -1.9640475 | 7.76E-20 | 1.46E-17 |
|  | | T17H7.7 | | 36.0232509 | -1.9556607 | 3.59E-07 | 1.34E-05 |
|  | | T22H6.2 | | 67.6879525 | -1.9464742 | 1.95E-14 | 2.23E-12 |
|  | | F26A1.9 | | 18.1764574 | -1.9324819 | 0.000194 | 0.00392034 |
|  | | F28D1.9 | | 24.4205116 | -1.927035 | 4.69E-06 | 0.00014307 |
|  | | F10F2.3 | | 34.6591359 | -1.9268357 | 4.94E-08 | 2.31E-06 |
|  | | K07E3.9 | | 25.0953491 | -1.9185863 | 2.06E-06 | 6.68E-05 |
|  | | Y47D3B.10 | | 64.2043024 | -1.9158022 | 3.21E-13 | 3.10E-11 |
|  | | Y48G8AL.16 | | 25.7970449 | -1.9120535 | 0.00030723 | 0.00586224 |
|  | | C56C10.4 | | 17.1713255 | -1.9099267 | 7.78E-05 | 0.00175859 |
|  | | Y47G6A.15 | | 690.261159 | -1.9086766 | 3.29E-08 | 1.62E-06 |
|  | | C26B9.3 | | 62.382541 | -1.9083592 | 1.79E-12 | 1.59E-10 |
|  | | Y75B8A.28 | | 26.4418888 | -1.9029923 | 2.25E-05 | 0.00057673 |
|  | | T01B7.13 | | 48.948317 | -1.9016767 | 5.05E-05 | 0.00119232 |
|  | | C29F9.2 | | 47.2752933 | -1.89664 | 1.28E-06 | 4.30E-05 |
|  | | ZC443.3 | | 17.0085688 | -1.89243 | 0.0001166 | 0.00250392 |
|  | | T22B11.2 | | 13.8667072 | -1.8860162 | 0.00038811 | 0.00707111 |
|  | | F13H8.5 | | 52.3579975 | -1.8847711 | 6.62E-10 | 4.48E-08 |
|  | | C08E8.4 | | 15.3507753 | -1.8794596 | 0.00022827 | 0.0045239 |
|  | | F13D11.4 | | 28.3862967 | -1.8784991 | 8.95E-07 | 3.10E-05 |
|  | | F33A8.7 | | 47.5556196 | -1.8778815 | 1.60E-09 | 1.01E-07 |
|  | | F49H12.5 | | 109.376443 | -1.873068 | 3.17E-14 | 3.38E-12 |
|  | | T23G11.6 | | 22.9330992 | -1.8721131 | 1.39E-05 | 0.00037429 |
|  | | T10E9.3 | | 84.001767 | -1.8700704 | 3.52E-15 | 4.32E-13 |
|  | | F52D1.1 | | 36.4596237 | -1.8603873 | 1.35E-07 | 5.63E-06 |
|  | | C31C9.2 | | 85.0147482 | -1.858836 | 1.30E-16 | 1.93E-14 |
|  | | K08C9.4 | | 156.817808 | -1.8376096 | 1.02E-24 | 2.61E-22 |
|  | | C42D4.13 | | 22.4765732 | -1.8345193 | 0.00017377 | 0.00355598 |
|  | | C05G5.6 | | 22.4508082 | -1.8328048 | 3.28E-05 | 0.00081688 |
|  | | C09E8.2 | | 20.1637132 | -1.8283118 | 0.00027463 | 0.00532119 |
|  | | R74.2 | | 30.5133712 | -1.8228032 | 5.36E-06 | 0.00015817 |
|  | | C50F2.6 | | 46.8486665 | -1.8213131 | 1.98E-08 | 1.01E-06 |
|  | | D1086.3 | | 45.2391078 | -1.8158113 | 3.42E-09 | 2.08E-07 |
|  | | R07E5.4 | | 74.125117 | -1.8150171 | 2.27E-13 | 2.23E-11 |
|  | | ZK377.1 | | 38.5033961 | -1.8126141 | 3.92E-06 | 0.00012158 |
|  | | F10D11.6 | | 90.1507591 | -1.8090904 | 3.06E-15 | 3.84E-13 |
|  | | T19C3.2 | | 130.664963 | -1.8072636 | 3.13E-13 | 3.05E-11 |
|  | | T06A4.3 | | 25.756539 | -1.8013947 | 5.16E-06 | 0.00015503 |
|  | | R09B5.9 | | 45.583448 | -1.7996661 | 3.70E-08 | 1.78E-06 |
|  | | BE10.2 | | 26.4140981 | -1.7959943 | 3.66E-06 | 0.00011455 |
|  | | F41F3.3 | | 1928.00082 | -1.7937549 | 3.99E-26 | 1.16E-23 |
|  | | C29E4.1 | | 61.5470173 | -1.793631 | 2.57E-11 | 2.03E-09 |
|  | | T19B10.2 | | 235.934488 | -1.7934346 | 6.23E-30 | 2.20E-27 |
|  | | C05C8.3 | | 163.768536 | -1.7894673 | 5.52E-20 | 1.11E-17 |
|  | | F26B1.4 | | 40.7686747 | -1.7815308 | 1.88E-08 | 9.73E-07 |
|  | | C04G6.2 | | 87.7209406 | -1.7736288 | 2.97E-09 | 1.81E-07 |
|  | | F32A5.4 | | 63.0129571 | -1.772234 | 7.09E-10 | 4.77E-08 |
|  | | C06G1.1 | | 35.4961646 | -1.772201 | 1.65E-07 | 6.65E-06 |
|  | | C28H8.5 | | 18.8323179 | -1.7715869 | 0.00024475 | 0.0048081 |
|  | | Y65B4BR.6 | | 383.486516 | -1.7700589 | 2.31E-14 | 2.53E-12 |
|  | | H10E21.4 | | 241.343821 | -1.7674755 | 4.19E-16 | 5.98E-14 |
|  | | F32A11.6 | | 29.5638104 | -1.7634431 | 5.31E-06 | 0.00015799 |
|  | | C29F3.2 | | 18.66728 | -1.7551343 | 0.00029259 | 0.00564982 |
|  | | T22B7.3 | | 18.644783 | -1.7535095 | 0.0001585 | 0.00327309 |
|  | | F37A8.5 | | 15.0183327 | -1.7483475 | 0.00053634 | 0.0092779 |
|  | | C30H6.5 | | 27.0849361 | -1.7416477 | 1.43E-05 | 0.00038283 |
|  | | ZK180.5 | | 294.624256 | -1.7344538 | 1.24E-11 | 1.02E-09 |
|  | | Y62E10A.19 | | 21.9536562 | -1.7299087 | 5.12E-05 | 0.00120136 |
|  | | F15B9.8 | | 137.360953 | -1.7294566 | 2.70E-18 | 4.48E-16 |
|  | | C06B3.3 | | 43.8847087 | -1.7291202 | 6.39E-08 | 2.91E-06 |
|  | | F38A6.4 | | 20.9698226 | -1.7049504 | 0.00019393 | 0.00392034 |
|  | | F45G2.5 | | 21.6380443 | -1.7025215 | 0.00022963 | 0.00454283 |
|  | | Y42H9B.1 | | 21.6200048 | -1.7011634 | 6.22E-05 | 0.00144031 |
|  | | C02E7.6 | | 228.683406 | -1.7005097 | 1.03E-13 | 1.02E-11 |
|  | | F53F1.4 | | 1139.0435 | -1.6932091 | 3.81E-34 | 1.87E-31 |
|  | | R11A5.7 | | 63.8885586 | -1.6928554 | 7.16E-11 | 5.40E-09 |
|  | | K12G11.3 | | 40.243292 | -1.691733 | 1.09E-06 | 3.70E-05 |
|  | | M195.1 | | 881.520789 | -1.6905782 | 3.68E-20 | 7.56E-18 |
|  | | F52E4.5 | | 20.7960991 | -1.6896194 | 8.25E-05 | 0.0018459 |
|  | | C38C6.6 | | 130.518662 | -1.6822072 | 7.22E-10 | 4.82E-08 |
|  | | T24F1.7 | | 28.237106 | -1.6770646 | 1.17E-05 | 0.00032644 |
|  | | F22D6.10 | | 316.089122 | -1.6730028 | 1.08E-34 | 5.54E-32 |
|  | | K01D12.9 | | 75.4322723 | -1.6691187 | 1.38E-11 | 1.12E-09 |
|  | | B0213.5 | | 44.1305621 | -1.6497537 | 5.70E-05 | 0.00132465 |
|  | | ZK154.1 | | 48.1668247 | -1.6488957 | 7.37E-08 | 3.29E-06 |
|  | | C15C6.1 | | 44.5633014 | -1.6395788 | 4.76E-08 | 2.26E-06 |
|  | | C07E3.10 | | 21.4741176 | -1.6277726 | 0.00055059 | 0.00950984 |
|  | | Y66A7A.6 | | 77.0310075 | -1.6247581 | 3.09E-10 | 2.14E-08 |
|  | | Y11D7A.11 | | 321.448119 | -1.624003 | 8.79E-24 | 2.03E-21 |
|  | | C29F7.2 | | 59.4371841 | -1.6185144 | 2.42E-09 | 1.52E-07 |
|  | | C33G3.3 | | 51.3570986 | -1.6161038 | 1.52E-07 | 6.26E-06 |
|  | | C05E4.9 | | 275.483716 | -1.607015 | 9.47E-15 | 1.13E-12 |
|  | | C29F3.5 | | 35.8375209 | -1.6064108 | 9.78E-06 | 0.0002763 |
|  | | T02C1.1 | | 34.3749736 | -1.598679 | 0.00024056 | 0.00474235 |
|  | | Y48G8AL.12 | | 1002.39658 | -1.5983936 | 3.25E-13 | 3.11E-11 |
|  | | C03G6.15 | | 66.6751362 | -1.5966119 | 1.23E-10 | 9.00E-09 |
|  | | C05E7.2 | | 118.802913 | -1.5855965 | 1.11E-16 | 1.69E-14 |
|  | | F38B2.6 | | 52.8641286 | -1.5743127 | 1.26E-05 | 0.00034436 |
|  | | Y47D7A.15 | | 703.06823 | -1.5715327 | 1.17E-49 | 1.20E-46 |
|  | | C05E7.1 | | 67.2131173 | -1.5555181 | 1.56E-07 | 6.35E-06 |
|  | | F15E6.2 | | 29.0299904 | -1.5524936 | 3.50E-05 | 0.00085923 |
|  | | R02E4.3 | | 234.092334 | -1.5516295 | 2.85E-05 | 0.00071595 |
|  | | M153.1 | | 88.3492733 | -1.5372002 | 4.44E-09 | 2.65E-07 |
|  | | F09F7.6 | | 62.7197628 | -1.5168461 | 5.38E-09 | 3.16E-07 |
|  | | R05H5.8 | | 62.9090375 | -1.5029588 | 9.29E-08 | 4.03E-06 |
|  | | C01B10.11 | | 64.6614601 | -1.4994396 | 1.18E-08 | 6.30E-07 |
|  | | K08C7.2 | | 31.3859094 | -1.4984813 | 0.00021579 | 0.00431427 |
|  | | T21C9.9 | | 36.9581163 | -1.4950075 | 3.08E-06 | 9.76E-05 |
|  | | F32B5.8 | | 433.43995 | -1.4947462 | 3.54E-16 | 5.12E-14 |
|  | | Y57G11B.2 | | 32.2052738 | -1.4722656 | 0.00011585 | 0.00249265 |
|  | | C36E6.8 | | 46.8682544 | -1.4642493 | 5.38E-07 | 1.93E-05 |
|  | | B0334.13 | | 43.4212076 | -1.4476496 | 6.27E-06 | 0.00018201 |
|  | | W05G11.6 | | 90.4433257 | -1.446222 | 2.30E-11 | 1.83E-09 |
|  | | C40H1.4 | | 25.5882702 | -1.440023 | 0.00026541 | 0.00515129 |
|  | | F25H8.5 | | 22.4321534 | -1.4309281 | 0.0004521 | 0.00806782 |
|  | | C02E7.7 | | 283.572618 | -1.4255602 | 4.51E-18 | 7.38E-16 |
|  | | C34H4.4 | | 708.230195 | -1.4141225 | 2.41E-06 | 7.73E-05 |
|  | | M88.6 | | 27.0428124 | -1.4135942 | 0.00036057 | 0.00666611 |
|  | | F49C12.7 | | 23.431549 | -1.4127989 | 0.00034459 | 0.0064446 |
|  | | D2085.1 | | 36.6423169 | -1.4121319 | 1.50E-05 | 0.00039807 |
|  | | W05G11.3 | | 410.61674 | -1.4024261 | 4.60E-14 | 4.68E-12 |
|  | | R12E2.15 | | 464.211721 | -1.3914221 | 3.60E-07 | 1.34E-05 |
|  | | R02F11.1 | | 51.7118265 | -1.3910418 | 0.0002364 | 0.00466855 |
|  | | C34D4.15 | | 68.8517805 | -1.3882602 | 6.75E-07 | 2.40E-05 |
|  | | R02E12.6 | | 36.1497108 | -1.3851889 | 2.88E-05 | 0.00072259 |
|  | | R12E2.14 | | 511.906615 | -1.3850757 | 1.00E-07 | 4.32E-06 |
|  | | C07D10.4 | | 57.4687516 | -1.3846289 | 1.19E-05 | 0.00032987 |
|  | | Y110A2AL.8 | | 24.2637263 | -1.3823442 | 0.00049691 | 0.0087296 |
|  | | C49F8.3 | | 150.766759 | -1.3809845 | 1.20E-14 | 1.41E-12 |
|  | | F23B2.5 | | 38.2597195 | -1.3719766 | 7.75E-05 | 0.00175761 |
|  | | F53F4.5 | | 67.6199817 | -1.3700406 | 5.43E-08 | 2.52E-06 |
|  | | C29F4.1 | | 951.202962 | -1.3496719 | 4.88E-13 | 4.64E-11 |
|  | | M01H9.1 | | 43.0789201 | -1.3496529 | 1.39E-05 | 0.00037429 |
|  | | F44A6.5 | | 80.7373892 | -1.3449953 | 5.02E-07 | 1.81E-05 |
|  | | R12E2.7 | | 726.550196 | -1.3258912 | 7.67E-08 | 3.40E-06 |
|  | | K10B3.1 | | 41.4113176 | -1.3255711 | 1.70E-05 | 0.00044119 |
|  | | T28F4.5 | | 503.158464 | -1.3203962 | 2.54E-09 | 1.59E-07 |
|  | | F26G1.5 | | 106.119535 | -1.3198702 | 2.36E-10 | 1.65E-08 |
|  | | C52G5.2 | | 121.153324 | -1.3027821 | 1.30E-09 | 8.45E-08 |
|  | | Y2H9A.3 | | 629.296764 | -1.2962846 | 7.31E-13 | 6.66E-11 |
|  | | F55C10.2 | | 294.981456 | -1.2740346 | 7.04E-12 | 5.98E-10 |
|  | | T03F1.11 | | 71.6861959 | -1.2712209 | 0.00056024 | 0.00966186 |
|  | | F38A3.2 | | 807.80157 | -1.2691405 | 4.24E-07 | 1.55E-05 |
|  | | F46E10.9 | | 132.539469 | -1.2601475 | 1.15E-07 | 4.91E-06 |
|  | | F42F12.9 | | 65.1563056 | -1.2598783 | 4.43E-07 | 1.61E-05 |
|  | | F47B10.7 | | 103.864891 | -1.2542556 | 1.05E-05 | 0.00029401 |
|  | | C26F1.1 | | 51.3245998 | -1.2412478 | 1.66E-05 | 0.00043223 |
|  | | R12C12.9 | | 52.6475609 | -1.2281189 | 1.32E-05 | 0.00035889 |
|  | | F08G5.4 | | 376.94242 | -1.2204121 | 2.84E-05 | 0.00071595 |
|  | | C08F11.13 | | 40.9417043 | -1.2186374 | 0.00038355 | 0.00701071 |
|  | | D1025.4 | | 195.958302 | -1.2185793 | 7.92E-08 | 3.50E-06 |
|  | | M02D8.4 | | 69.4984787 | -1.2074928 | 1.50E-05 | 0.00039807 |
|  | | D1009.1 | | 50.4906172 | -1.2074471 | 1.05E-05 | 0.00029537 |
|  | | F54C9.3 | | 260.242237 | -1.2004434 | 5.25E-06 | 0.00015686 |
|  | | C15C7.5 | | 191.978668 | -1.1993858 | 5.00E-08 | 2.33E-06 |
|  | | T01D1.3 | | 42.0783345 | -1.194307 | 9.22E-05 | 0.00203513 |
|  | | F48E8.1 | | 38.2595268 | -1.1923314 | 0.0001238 | 0.00263865 |
|  | | ZK1307.1 | | 113.525282 | -1.188732 | 4.55E-09 | 2.70E-07 |
|  | | F45E1.4 | | 41.9010223 | -1.1856756 | 5.57E-05 | 0.0012963 |
|  | | B0222.6 | | 466.563328 | -1.1772035 | 2.58E-12 | 2.26E-10 |
|  | | F45D3.4 | | 88.5866772 | -1.1593579 | 8.66E-08 | 3.79E-06 |
|  | | T10E10.6 | | 42.8753211 | -1.1551147 | 8.37E-05 | 0.00186905 |
|  | | C28H8.11 | | 118.971436 | -1.1525675 | 5.85E-08 | 2.70E-06 |
|  | | F42G9.2 | | 82.7785649 | -1.1468877 | 5.70E-07 | 2.04E-05 |
|  | | C56A3.2 | | 216.134962 | -1.1310716 | 8.27E-12 | 6.92E-10 |
|  | | T28C6.6 | | 68.807334 | -1.1224801 | 5.01E-06 | 0.00015183 |
|  | | ZK270.1 | | 40.1060181 | -1.1206645 | 0.00057974 | 0.00992856 |
|  | | F42F12.6 | | 43.2152007 | -1.1202528 | 0.00014991 | 0.00312426 |
|  | | T01D1.4 | | 86.2642974 | -1.1162229 | 1.35E-07 | 5.63E-06 |
|  | | F42F12.7 | | 87.7007936 | -1.1134983 | 5.36E-06 | 0.00015817 |
|  | | W03A5.7 | | 53.9717215 | -1.1130712 | 0.00017301 | 0.00354685 |
|  | | T28C6.4 | | 97.5285421 | -1.1123572 | 9.14E-07 | 3.16E-05 |
|  | | F41E6.2 | | 647.859562 | -1.1039144 | 1.46E-08 | 7.69E-07 |
|  | | F09G8.6 | | 252.508149 | -1.1027512 | 1.06E-08 | 5.77E-07 |
|  | | C25B8.3 | | 710.922968 | -1.0978177 | 3.39E-12 | 2.92E-10 |
|  | | F48D6.4 | | 109.95508 | -1.0920068 | 9.68E-05 | 0.0021197 |
|  | | Y47D7A.13 | | 511.38338 | -1.090772 | 3.00E-05 | 0.00075022 |
|  | | T01B10.2 | | 323.428282 | -1.0904196 | 7.25E-11 | 5.42E-09 |
|  | | T25G12.5 | | 62.7336108 | -1.0762427 | 0.00057166 | 0.00981374 |
|  | | T18H9.1 | | 54.4598253 | -1.0718332 | 0.00017194 | 0.00353136 |
|  | | B0218.2 | | 75.6632748 | -1.0533631 | 2.39E-05 | 0.00060992 |
|  | | ZK909.3 | | 168.437736 | -1.0525029 | 3.81E-09 | 2.29E-07 |
|  | | F26H9.5 | | 53.415377 | -1.0311099 | 0.00019544 | 0.00394234 |
|  | | T03E6.7 | | 774.222361 | -1.0236601 | 3.02E-17 | 4.80E-15 |
|  | | B0222.7 | | 264.876648 | -1.021042 | 7.64E-15 | 9.18E-13 |
|  | | Y51H4A.9 | | 63.9671941 | -1.0166059 | 4.53E-05 | 0.00108825 |
|  | | B0222.8 | | 244.505746 | -1.0165055 | 1.56E-09 | 9.96E-08 |
|  | | F09B12.3 | | 78.1741924 | -1.0115084 | 3.43E-06 | 0.00010821 |
|  | | T08G2.3 | | 92.9217634 | -1.0090527 | 0.00012054 | 0.0025838 |
|  | | W05H9.3 | | 184.300304 | -1.0084155 | 4.91E-08 | 2.31E-06 |
|  | | C50B6.4 | | 894.438219 | -0.9993621 | 2.93E-08 | 1.47E-06 |
|  | | W03A5.8 | | 116.807882 | -0.9944499 | 0.00050115 | 0.00879034 |
|  | | F42F12.1 | | 89.8723334 | -0.9837831 | 1.95E-06 | 6.36E-05 |
|  | | C34E7.4 | | 132.635277 | -0.9719069 | 2.59E-07 | 9.99E-06 |
|  | | T10E10.1 | | 74.1754267 | -0.953546 | 0.00014003 | 0.00293773 |
|  | | T02C5.1 | | 207.725578 | -0.9392502 | 7.91E-07 | 2.77E-05 |
|  | | C15C8.3 | | 173.771043 | -0.9156775 | 1.65E-08 | 8.59E-07 |
|  | | T08A9.12 | | 123.542873 | -0.9147339 | 2.41E-06 | 7.73E-05 |
|  | | F35B3.4 | | 126.854709 | -0.8900797 | 7.13E-06 | 0.00020378 |
|  | | T28H10.3 | | 138.606321 | -0.883352 | 0.00014018 | 0.00293773 |
|  | | F52H3.5 | | 71.4150201 | -0.8547272 | 0.00022449 | 0.00446451 |
|  | | Y97E10C.1 | | 133.253167 | -0.8331873 | 6.74E-06 | 0.00019376 |
|  | | VZK822L.1 | | 127.864797 | -0.8266903 | 0.00038073 | 0.00698174 |
|  | | T07C4.1 | | 85.9301366 | -0.824843 | 0.00016316 | 0.00335701 |
|  | | DH11.2 | | 118.125136 | -0.8231491 | 0.00032409 | 0.00610146 |
|  | | Y69F12A.2 | | 81.811668 | -0.8115379 | 0.00038587 | 0.00704171 |
|  | | C44C10.1 | | 201.36446 | -0.8091435 | 2.83E-07 | 1.08E-05 |
|  | | VC5.3 | | 304.720835 | -0.8087486 | 3.03E-05 | 0.00075601 |
|  | | M03A8.1 | | 195.160945 | -0.7946305 | 7.93E-05 | 0.00178036 |
|  | | F09B9.3 | | 84.0823217 | -0.7878186 | 0.00017767 | 0.00362273 |
|  | | R07E3.1 | | 130.933322 | -0.7624536 | 2.05E-05 | 0.00052866 |
|  | | F25B5.3 | | 267.230336 | -0.7554695 | 7.21E-09 | 4.13E-07 |
|  | | F09B9.4 | | 95.9719017 | -0.7409749 | 0.0003124 | 0.00593088 |
|  | | Y37D8A.16 | | 95.7925118 | -0.7367025 | 0.00022046 | 0.00439977 |
|  | | T02E1.5 | | 134.255729 | -0.7210615 | 0.00035501 | 0.00660665 |
|  | | C07A12.4 | | 528.183271 | -0.712862 | 1.28E-08 | 6.79E-07 |
|  | | D1037.3 | | 398.756648 | -0.6304393 | 2.16E-08 | 1.11E-06 |
|  | | C28H8.14 | | 915.804778 | -0.591436 | 0.00039299 | 0.00712659 |
|  | | F44F4.11 | | 244.531884 | -0.5858578 | 0.00033625 | 0.0063095 |
|  | | C55B7.4 | | 2314.44122 | -0.5754033 | 0.00045128 | 0.00806583 |
|  | | C31E10.7 | | 166.987186 | -0.5742277 | 0.00036673 | 0.00673582 |
|  | | F31C3.1 | | 624.971731 | -0.554804 | 0.0004646 | 0.00822595 |
|  | | T04G9.5 | | 303.181296 | -0.5339066 | 0.00025129 | 0.00491193 |
|  | | F07A5.7 | | 364.811275 | -0.5244904 | 0.00035096 | 0.0065419 |
|  | | F55C10.3 | | 269.517471 | -0.5199915 | 0.00010568 | 0.00229131 |
|  | | H06O01.1 | | 529.441139 | -0.5089063 | 1.62E-06 | 5.39E-05 |
|  | | F57H12.1 | | 330.391347 | -0.4272104 | 0.00052442 | 0.00911353 |
|  | | F07H5.5 | | 697.114285 | -0.4112054 | 1.70E-05 | 0.00044119 |
|  | | C54G6.5 | | 664.404388 | -0.3581705 | 0.0001873 | 0.00379853 |
| **Up-regulation** | **Gene ID** | | **BaseMean** | | **log2FoldChange** | **pvalue** | **padj** |
|  | C35D10.11 | | 396.6231 | | 0.491686 | 8.53E-05 | 0.001897 |
|  | R05H5.3 | | 189.8737 | | 0.603024 | 0.000122 | 0.002609 |
|  | C05C10.3 | | 240.8865 | | 0.609101 | 1.23E-05 | 0.000339 |
|  | F09E10.3 | | 188.132 | | 0.621269 | 0.000361 | 0.006666 |
|  | C49F5.1 | | 273.4277 | | 0.628346 | 0.000526 | 0.009121 |
|  | F53H4.2 | | 211.0046 | | 0.65201 | 9.61E-05 | 0.002108 |
|  | ZK1127.10 | | 274.1735 | | 0.654441 | 1.07E-06 | 3.64E-05 |
|  | R01E6.3 | | 157.0142 | | 0.659117 | 0.000158 | 0.003261 |
|  | F59B8.2 | | 300.001 | | 0.70697 | 1.22E-07 | 5.18E-06 |
|  | C37H5.6 | | 118.2945 | | 0.713895 | 9.00E-05 | 0.001993 |
|  | M02F4.8 | | 167.3492 | | 0.721544 | 5.23E-06 | 0.000157 |
|  | C02B10.1 | | 112.2055 | | 0.741339 | 4.84E-05 | 0.001155 |
|  | R09B3.2 | | 751.613 | | 0.74252 | 0.000243 | 0.004786 |
|  | C03C10.3 | | 103.1126 | | 0.826738 | 0.00036 | 0.006666 |
|  | R02D3.1 | | 66.94985 | | 0.826851 | 0.000407 | 0.007327 |
|  | ZC168.5 | | 87.87114 | | 0.856027 | 0.000154 | 0.003202 |
|  | K07F5.1 | | 368.1597 | | 0.862593 | 0.000382 | 0.006994 |
|  | F01F1.6 | | 75.17198 | | 0.867137 | 9.47E-05 | 0.002082 |
|  | C48E7.1 | | 115.7976 | | 0.88545 | 2.14E-06 | 6.92E-05 |
|  | D1086.10 | | 577.618 | | 0.886175 | 0.000151 | 0.003138 |
|  | C29F7.3 | | 138.3652 | | 0.891381 | 3.87E-07 | 1.43E-05 |
|  | T27C10.7 | | 109.0304 | | 0.892166 | 0.000112 | 0.002418 |
|  | C10H11.6 | | 114.5424 | | 0.894682 | 0.000414 | 0.007438 |
|  | R05F9.13 | | 599.1261 | | 0.927872 | 0.000123 | 0.002625 |
|  | F36H12.7 | | 339.3731 | | 0.930701 | 2.54E-05 | 0.000644 |
|  | M01E11.5 | | 439.972 | | 0.93237 | 6.91E-05 | 0.001582 |
|  | T06E4.6 | | 505.933 | | 0.932965 | 0.000295 | 0.00568 |
|  | ZK1251.6 | | 393.7536 | | 0.940184 | 0.000196 | 0.003946 |
|  | F15G9.1 | | 158.4307 | | 0.940975 | 1.49E-07 | 6.13E-06 |
|  | R05F9.8 | | 379.9763 | | 0.946183 | 1.36E-05 | 0.00037 |
|  | F13H8.7 | | 155.8088 | | 0.971689 | 4.98E-07 | 1.80E-05 |
|  | F32B6.6 | | 231.804 | | 0.972231 | 0.000521 | 0.009089 |
|  | W01F3.2 | | 54.62247 | | 0.981416 | 0.00025 | 0.004912 |
|  | F08H9.2 | | 88.95129 | | 0.981484 | 0.000131 | 0.002785 |
|  | ZK546.6 | | 351.0422 | | 0.982938 | 4.05E-06 | 0.000125 |
|  | ZC434.9 | | 69.44006 | | 0.997514 | 0.000328 | 0.006167 |
|  | F08F3.3 | | 122.095 | | 0.998802 | 4.47E-05 | 0.001076 |
|  | C34F11.6 | | 228.372 | | 0.999279 | 1.19E-05 | 0.000331 |
|  | C10C5.3 | | 55.26869 | | 1.007558 | 0.000105 | 0.002283 |
|  | C04G2.4 | | 348.1981 | | 1.011797 | 3.04E-05 | 0.000758 |
|  | T05H4.13 | | 139.5304 | | 1.023065 | 9.65E-06 | 0.000273 |
|  | T13F2.10 | | 105.8812 | | 1.036958 | 8.64E-05 | 0.001917 |
|  | K07F5.11 | | 218.4682 | | 1.039525 | 0.000181 | 0.003681 |
|  | ZK354.11 | | 290.3078 | | 1.048615 | 0.0001 | 0.002187 |
|  | T09F3.3 | | 61.97885 | | 1.053298 | 0.000296 | 0.005686 |
|  | F58A6.8 | | 291.3201 | | 1.055139 | 1.89E-06 | 6.21E-05 |
|  | T07C4.5 | | 870.8177 | | 1.06142 | 1.83E-07 | 7.26E-06 |
|  | C06A6.4 | | 185.4037 | | 1.066876 | 9.92E-08 | 4.28E-06 |
|  | C39B5.5 | | 280.3295 | | 1.068119 | 9.36E-09 | 5.18E-07 |
|  | F41E7.5 | | 384.6594 | | 1.069067 | 1.17E-06 | 3.95E-05 |
|  | K07H8.6 | | 1028.307 | | 1.096798 | 1.51E-05 | 0.000401 |
|  | Y87G2A.5 | | 165.2748 | | 1.099925 | 7.83E-12 | 6.60E-10 |
|  | Y43B11AR.1 | | 50.49427 | | 1.102525 | 7.15E-05 | 0.001631 |
|  | F59D8.1 | | 59.04919 | | 1.110596 | 1.24E-05 | 0.000341 |
|  | ZK354.4 | | 152.062 | | 1.127925 | 1.55E-05 | 0.000409 |
|  | C14B1.9 | | 53.75291 | | 1.131245 | 0.000362 | 0.006679 |
|  | K08F4.8 | | 108.8281 | | 1.136042 | 6.43E-06 | 0.000186 |
|  | T28H11.1 | | 214.6707 | | 1.143772 | 1.66E-05 | 0.000432 |
|  | Y45F10C.4 | | 224.143 | | 1.145441 | 8.98E-16 | 1.22E-13 |
|  | T28H11.5 | | 177.5201 | | 1.149601 | 5.06E-05 | 0.001192 |
|  | F13G11.3 | | 80.91524 | | 1.149609 | 4.12E-07 | 1.51E-05 |
|  | C24H10.5 | | 51.31578 | | 1.157892 | 3.32E-05 | 0.000825 |
|  | K03H1.4 | | 305.2019 | | 1.166058 | 7.12E-20 | 1.36E-17 |
|  | C09G5.4 | | 735.0639 | | 1.168315 | 1.18E-07 | 5.03E-06 |
|  | T28F3.4 | | 33.3823 | | 1.173878 | 0.000323 | 0.006096 |
|  | ZC477.1 | | 54.90105 | | 1.175081 | 0.000104 | 0.002269 |
|  | ZK354.1 | | 108.3315 | | 1.177906 | 6.30E-06 | 0.000183 |
|  | C14B1.2 | | 52.93522 | | 1.18173 | 3.50E-05 | 0.000859 |
|  | C34G6.2 | | 43.25579 | | 1.181906 | 5.56E-05 | 0.001296 |
|  | C09B9.6 | | 138.9114 | | 1.183699 | 7.43E-07 | 2.61E-05 |
|  | T05A1.2 | | 1789.599 | | 1.197634 | 5.18E-05 | 0.001214 |
|  | Y43E12A.1 | | 47.50539 | | 1.204884 | 0.000132 | 0.002786 |
|  | R13A5.6 | | 56.74827 | | 1.219764 | 3.47E-05 | 0.000859 |
|  | Y45F10C.3 | | 50.79395 | | 1.227238 | 2.39E-05 | 0.00061 |
|  | ZK354.5 | | 199.066 | | 1.230804 | 2.69E-07 | 1.03E-05 |
|  | Y77E11A.15 | | 1109.352 | | 1.240843 | 4.99E-06 | 0.000151 |
|  | F27C8.6 | | 67.37612 | | 1.263539 | 0.000357 | 0.006625 |
|  | W05B2.5 | | 1595.56 | | 1.269058 | 3.15E-07 | 1.19E-05 |
|  | C42D8.2 | | 914.9669 | | 1.273272 | 3.71E-08 | 1.78E-06 |
|  | T23G11.2 | | 34.6817 | | 1.287384 | 0.000264 | 0.00514 |
|  | F15A2.1 | | 902.8073 | | 1.288601 | 1.04E-06 | 3.54E-05 |
|  | F14B8.4 | | 33.06226 | | 1.289392 | 0.000394 | 0.007127 |
|  | C15A11.6 | | 420.9918 | | 1.302645 | 2.63E-19 | 4.63E-17 |
|  | H31G24.4 | | 63.10906 | | 1.30675 | 4.37E-05 | 0.001059 |
|  | ZK858.3 | | 42.73368 | | 1.319217 | 5.10E-05 | 0.001201 |
|  | F57B1.4 | | 1200.527 | | 1.334268 | 5.27E-07 | 1.90E-05 |
|  | T28H11.6 | | 40.76175 | | 1.336348 | 0.000202 | 0.004068 |
|  | EEED8.3 | | 30.91303 | | 1.337184 | 0.000109 | 0.002356 |
|  | Y105C5B.5 | | 67.92095 | | 1.343968 | 1.03E-06 | 3.52E-05 |
|  | T06E6.2 | | 87.09056 | | 1.3586 | 6.67E-05 | 0.001532 |
|  | W03G11.1 | | 1536.258 | | 1.358839 | 1.71E-06 | 5.67E-05 |
|  | F23H12.9 | | 37.16164 | | 1.359532 | 2.14E-05 | 0.000551 |
|  | T11F9.3 | | 46.21174 | | 1.367693 | 1.39E-05 | 0.000374 |
|  | W06B4.1 | | 26.62421 | | 1.367942 | 0.000504 | 0.008834 |
|  | T04G9.7 | | 65.77657 | | 1.368574 | 6.60E-08 | 2.99E-06 |
|  | C17E4.3 | | 27.28399 | | 1.3723 | 0.000263 | 0.005127 |
|  | W02A2.7 | | 69.99565 | | 1.375222 | 0.000464 | 0.00822 |
|  | Y73B6BL.38 | | 53.40273 | | 1.375275 | 0.000296 | 0.005684 |
|  | D1086.11 | | 148.3162 | | 1.378974 | 1.53E-14 | 1.76E-12 |
|  | F18A1.7 | | 73.95941 | | 1.383985 | 2.45E-07 | 9.59E-06 |
|  | F32A5.2 | | 77.13374 | | 1.38557 | 1.89E-07 | 7.50E-06 |
|  | Y75B12B.1 | | 82.68149 | | 1.392131 | 7.39E-10 | 4.91E-08 |
|  | M02F4.7 | | 33.05069 | | 1.396568 | 3.67E-05 | 0.000897 |
|  | F35C11.5 | | 33.68961 | | 1.401187 | 9.08E-05 | 0.002008 |
|  | F41F3.4 | | 2078.42 | | 1.407847 | 1.67E-07 | 6.70E-06 |
|  | F26F12.1 | | 1483.428 | | 1.408991 | 1.91E-08 | 9.84E-07 |
|  | M18.1 | | 1949.857 | | 1.411077 | 4.86E-07 | 1.76E-05 |
|  | C14F11.4 | | 93.19582 | | 1.425152 | 1.26E-10 | 9.16E-09 |
|  | F10C1.9 | | 30.59537 | | 1.430324 | 0.000563 | 0.0097 |
|  | C28C12.2 | | 67.86486 | | 1.433908 | 6.56E-06 | 0.000189 |
|  | C05C10.5 | | 30.39708 | | 1.462788 | 0.00035 | 0.006542 |
|  | K10B2.3 | | 60.47841 | | 1.47146 | 9.70E-08 | 4.20E-06 |
|  | C12D8.14 | | 46.87803 | | 1.471893 | 9.48E-05 | 0.002082 |
|  | F17C8.9 | | 25.6508 | | 1.475636 | 0.000184 | 0.003738 |
|  | Y45F10A.2 | | 55.20112 | | 1.485849 | 0.000473 | 0.008348 |
|  | F38A3.1 | | 1756.198 | | 1.488655 | 2.09E-10 | 1.48E-08 |
|  | T05G5.7 | | 58.50532 | | 1.488967 | 1.44E-07 | 5.99E-06 |
|  | F59D8.2 | | 57.37722 | | 1.491801 | 1.60E-08 | 8.39E-07 |
|  | DY3.8 | | 33.53134 | | 1.499109 | 9.43E-06 | 0.000268 |
|  | T08A9.11 | | 65.6032 | | 1.510799 | 7.59E-09 | 4.27E-07 |
|  | ZK783.2 | | 94.34688 | | 1.513934 | 6.74E-13 | 6.24E-11 |
|  | F43D9.4 | | 222.3251 | | 1.514883 | 3.38E-08 | 1.65E-06 |
|  | ZK1193.1 | | 1171.326 | | 1.515425 | 4.05E-11 | 3.14E-09 |
|  | F52E1.1 | | 104.8317 | | 1.521337 | 1.45E-07 | 6.01E-06 |
|  | T01C3.3 | | 70.01376 | | 1.529232 | 1.50E-09 | 9.62E-08 |
|  | C55C2.2 | | 23.8308 | | 1.534332 | 0.000137 | 0.002885 |
|  | C28D4.3 | | 63.25893 | | 1.540131 | 9.49E-07 | 3.27E-05 |
|  | F49E12.2 | | 19.39537 | | 1.541356 | 0.000478 | 0.008422 |
|  | C50B6.2 | | 94.63856 | | 1.54876 | 1.32E-06 | 4.45E-05 |
|  | F48E3.4 | | 46.85178 | | 1.553006 | 8.29E-08 | 3.64E-06 |
|  | F22B3.4 | | 72.1223 | | 1.554046 | 4.85E-05 | 0.001155 |
|  | T25E12.5 | | 48.14394 | | 1.555387 | 1.32E-05 | 0.000359 |
|  | C35B1.4 | | 70.51453 | | 1.560074 | 1.04E-09 | 6.76E-08 |
|  | C24F3.6 | | 2052.83 | | 1.561443 | 1.35E-11 | 1.10E-09 |
|  | F26D10.10 | | 36.64226 | | 1.566018 | 1.42E-05 | 0.000382 |
|  | C39D10.8 | | 31.56609 | | 1.57272 | 1.39E-05 | 0.000374 |
|  | T11F8.3 | | 47.47963 | | 1.582085 | 4.15E-05 | 0.001011 |
|  | C44B7.5 | | 103.057 | | 1.582722 | 3.40E-15 | 4.23E-13 |
|  | ZK228.4 | | 84.85712 | | 1.586147 | 2.05E-06 | 6.67E-05 |
|  | F53F8.4 | | 20.55141 | | 1.588088 | 0.000309 | 0.005874 |
|  | R06C7.4 | | 113.5436 | | 1.594087 | 4.06E-11 | 3.14E-09 |
|  | ZK228.3 | | 34.19554 | | 1.609946 | 4.66E-06 | 0.000143 |
|  | H02I12.5 | | 23.49116 | | 1.615718 | 0.000468 | 0.008272 |
|  | F54C9.8 | | 57.17688 | | 1.619582 | 2.48E-06 | 7.92E-05 |
|  | F11E6.3 | | 891.4317 | | 1.621386 | 3.58E-06 | 0.000112 |
|  | C15A11.5 | | 438.5931 | | 1.622042 | 8.55E-26 | 2.30E-23 |
|  | T12G3.6 | | 30.40835 | | 1.625737 | 5.74E-06 | 0.000168 |
|  | K09F5.2 | | 62.95011 | | 1.629442 | 2.33E-10 | 1.63E-08 |
|  | B0280.5 | | 209.6253 | | 1.630927 | 1.84E-06 | 6.07E-05 |
|  | C39D10.7 | | 57.02942 | | 1.635589 | 4.99E-09 | 2.95E-07 |
|  | F14F7.1 | | 65.4156 | | 1.641824 | 6.52E-11 | 4.98E-09 |
|  | C44B12.5 | | 210.8344 | | 1.645535 | 8.47E-19 | 1.45E-16 |
|  | W03C9.7 | | 30.06565 | | 1.649492 | 6.67E-05 | 0.001532 |
|  | W05F2.3 | | 52.08625 | | 1.654617 | 2.42E-07 | 9.49E-06 |
|  | F49E12.1 | | 35.65939 | | 1.654817 | 8.64E-07 | 3.00E-05 |
|  | F16B4.4 | | 407.161 | | 1.671035 | 8.45E-16 | 1.16E-13 |
|  | B0454.5 | | 22.1936 | | 1.673985 | 0.000135 | 0.002842 |
|  | H02I12.1 | | 55.36351 | | 1.674041 | 3.46E-06 | 0.000109 |
|  | C45G9.12 | | 24.98947 | | 1.675756 | 4.91E-05 | 0.001165 |
|  | C25A1.8 | | 126.8381 | | 1.676729 | 2.85E-08 | 1.44E-06 |
|  | F14H3.6 | | 31.21961 | | 1.677279 | 5.13E-06 | 0.000155 |
|  | C07G2.1 | | 75.90963 | | 1.685149 | 1.09E-08 | 5.91E-07 |
|  | F09D12.1 | | 27.94806 | | 1.687598 | 6.67E-05 | 0.001532 |
|  | B0244.8 | | 64.4 | | 1.695381 | 3.59E-07 | 1.34E-05 |
|  | Y51F10.2 | | 32.36272 | | 1.704866 | 2.81E-05 | 0.000711 |
|  | C44B12.1 | | 218.5772 | | 1.712836 | 3.64E-27 | 1.14E-24 |
|  | Y48A6B.7 | | 73.00363 | | 1.713546 | 3.35E-07 | 1.26E-05 |
|  | C50H2.10 | | 47.03088 | | 1.722979 | 3.42E-05 | 0.000848 |
|  | W02F12.3 | | 19.22201 | | 1.726257 | 0.000203 | 0.004074 |
|  | F38A5.12 | | 29.26892 | | 1.726472 | 0.000259 | 0.005053 |
|  | C14F11.5 | | 43.56163 | | 1.729525 | 4.45E-08 | 2.12E-06 |
|  | C12D8.6 | | 27.95176 | | 1.734417 | 0.000143 | 0.002995 |
|  | F17E9.4 | | 115.4168 | | 1.734785 | 3.15E-08 | 1.56E-06 |
|  | ZC412.7 | | 72.97609 | | 1.752419 | 0.000138 | 0.002908 |
|  | T13F3.6 | | 318.8179 | | 1.783456 | 1.80E-07 | 7.17E-06 |
|  | ZC513.6 | | 48.13336 | | 1.800342 | 1.26E-07 | 5.31E-06 |
|  | C50H2.12 | | 55.73473 | | 1.801705 | 2.57E-06 | 8.20E-05 |
|  | K08D12.6 | | 410.9538 | | 1.819579 | 8.29E-06 | 0.000236 |
|  | C25A8.4 | | 24.81187 | | 1.826958 | 5.27E-06 | 0.000157 |
|  | T06E4.4 | | 769.3717 | | 1.857929 | 3.35E-10 | 2.30E-08 |
|  | H13N06.6 | | 29.08825 | | 1.859331 | 7.00E-07 | 2.47E-05 |
|  | T21E8.1 | | 35.99308 | | 1.859725 | 6.18E-08 | 2.83E-06 |
|  | F31F6.6 | | 13.80335 | | 1.862937 | 0.000461 | 0.008216 |
|  | C05D9.9 | | 30.73553 | | 1.865295 | 1.77E-06 | 5.85E-05 |
|  | T25B9.1 | | 29.41935 | | 1.879575 | 7.29E-07 | 2.57E-05 |
|  | C16E9.1 | | 17.9213 | | 1.889203 | 0.000463 | 0.00822 |
|  | C07G3.2 | | 17.91674 | | 1.889814 | 0.000251 | 0.004912 |
|  | F43C11.3 | | 137.9579 | | 1.897752 | 1.63E-05 | 0.000427 |
|  | T01B11.5 | | 16.4367 | | 1.899188 | 0.000175 | 0.003575 |
|  | C34F6.2 | | 1017.215 | | 1.906348 | 2.50E-22 | 5.34E-20 |
|  | K10C2.8 | | 29.9206 | | 1.908977 | 7.01E-06 | 0.000201 |
|  | Y69H2.14 | | 103.0313 | | 1.965195 | 5.87E-19 | 1.02E-16 |
|  | F57C2.4 | | 44.3675 | | 1.988079 | 3.71E-10 | 2.54E-08 |
|  | B0513.4 | | 42.72155 | | 1.988215 | 2.75E-09 | 1.69E-07 |
|  | F38A5.14 | | 33.03959 | | 1.995855 | 1.54E-06 | 5.15E-05 |
|  | ZK669.3 | | 30.74548 | | 2.004477 | 0.000151 | 0.003138 |
|  | W05E7.1 | | 57.36269 | | 2.00763 | 3.64E-09 | 2.20E-07 |
|  | C04F6.1 | | 235.6483 | | 2.018421 | 1.10E-31 | 4.95E-29 |
|  | F11G11.11 | | 1122.265 | | 2.019857 | 6.58E-26 | 1.86E-23 |
|  | C49F5.7 | | 161.939 | | 2.020784 | 1.16E-05 | 0.000325 |
|  | D1054.10 | | 299.9028 | | 2.027264 | 2.38E-27 | 7.67E-25 |
|  | C45B2.1 | | 483.7906 | | 2.039009 | 1.95E-07 | 7.69E-06 |
|  | F56B3.1 | | 74.43946 | | 2.040136 | 2.03E-14 | 2.29E-12 |
|  | Y57G11B.5 | | 84.13389 | | 2.048606 | 3.44E-17 | 5.40E-15 |
|  | Y41C4A.19 | | 44.3643 | | 2.090555 | 4.81E-11 | 3.70E-09 |
|  | F55B11.2 | | 159.38 | | 2.092428 | 4.74E-27 | 1.45E-24 |
|  | F57A8.8 | | 29.75076 | | 2.098518 | 9.17E-06 | 0.000261 |
|  | C05A9.1 | | 32.04824 | | 2.130029 | 2.62E-07 | 1.01E-05 |
|  | T04F8.8 | | 289.2336 | | 2.150432 | 8.42E-14 | 8.50E-12 |
|  | Y48G1C.13 | | 14.62564 | | 2.176905 | 0.00016 | 0.003305 |
|  | C34F6.3 | | 309.4101 | | 2.179544 | 2.57E-24 | 6.18E-22 |
|  | T05A10.5 | | 34.84407 | | 2.181294 | 9.70E-07 | 3.33E-05 |
|  | T12B3.2 | | 11.00761 | | 2.184376 | 0.000506 | 0.008856 |
|  | Y47G6A.33 | | 156.5851 | | 2.190859 | 2.07E-22 | 4.59E-20 |
|  | Y43F8C.2 | | 69.67811 | | 2.203635 | 2.59E-07 | 9.99E-06 |
|  | B0024.1 | | 174.9794 | | 2.22837 | 3.10E-30 | 1.17E-27 |
|  | F18E9.3 | | 14.13429 | | 2.229237 | 0.000318 | 0.006018 |
|  | F42A8.1 | | 44.01484 | | 2.297971 | 4.16E-12 | 3.56E-10 |
|  | B0213.2 | | 64.58598 | | 2.346932 | 3.27E-07 | 1.24E-05 |
|  | T21C9.3 | | 76.23425 | | 2.362022 | 2.72E-15 | 3.46E-13 |
|  | R04D3.3 | | 9.36275 | | 2.403564 | 0.000523 | 0.009097 |
|  | B0213.3 | | 42.23467 | | 2.424719 | 2.75E-06 | 8.75E-05 |
|  | K07A1.6 | | 49.45596 | | 2.457276 | 6.05E-10 | 4.12E-08 |
|  | Y38E10A.13 | | 22.67303 | | 2.463802 | 7.51E-05 | 0.001707 |
|  | C09G5.5 | | 955.2756 | | 2.483673 | 7.07E-13 | 6.49E-11 |
|  | C46C2.5 | | 13.14302 | | 2.490015 | 4.90E-05 | 0.001165 |
|  | C04F5.7 | | 11.16912 | | 2.524151 | 0.000104 | 0.002266 |
|  | D1086.6 | | 56.01481 | | 2.562808 | 1.15E-15 | 1.53E-13 |
|  | Y105C5A.8 | | 17.57734 | | 2.60397 | 3.06E-06 | 9.71E-05 |
|  | Y43C5A.7 | | 10.67713 | | 2.624438 | 0.000228 | 0.004524 |
|  | K02G10.7 | | 80.98486 | | 2.630691 | 4.73E-16 | 6.59E-14 |
|  | Y62H9A.5 | | 189.7248 | | 2.633682 | 1.46E-21 | 3.05E-19 |
|  | Y62H9A.4 | | 75.23883 | | 2.634822 | 5.03E-13 | 4.74E-11 |
|  | F55B11.3 | | 39.59063 | | 2.694276 | 1.05E-10 | 7.76E-09 |
|  | Y62H9A.6 | | 161.1484 | | 2.696483 | 1.53E-15 | 2.01E-13 |
|  | K11D12.13 | | 21.51552 | | 2.733549 | 3.39E-08 | 1.65E-06 |
|  | C53B4.5 | | 816.478 | | 2.760152 | 2.66E-25 | 7.00E-23 |
|  | F59A6.12 | | 24.63993 | | 2.772637 | 2.60E-08 | 1.32E-06 |
|  | D1054.11 | | 205.6592 | | 2.777062 | 7.24E-15 | 8.79E-13 |
|  | Y62H9A.3 | | 55.19097 | | 2.794328 | 4.31E-14 | 4.46E-12 |
|  | Y37D8A.19 | | 166.2328 | | 2.798903 | 1.18E-12 | 1.06E-10 |
|  | ZK813.7 | | 125.1636 | | 2.815072 | 9.44E-16 | 1.27E-13 |
|  | ZC373.2 | | 169.5124 | | 2.818097 | 5.49E-13 | 5.13E-11 |
|  | H01M10.3 | | 17.57097 | | 2.842204 | 2.85E-07 | 1.08E-05 |
|  | Y38E10A.26 | | 8.212191 | | 2.86197 | 0.000479 | 0.008425 |
|  | ZK813.2 | | 45.01307 | | 2.903276 | 2.56E-07 | 9.95E-06 |
|  | W02D9.6 | | 11.98478 | | 3.010365 | 1.43E-05 | 0.000383 |
|  | ZK813.3 | | 103.6273 | | 3.012498 | 6.48E-20 | 1.26E-17 |
|  | T15B7.4 | | 150.7548 | | 3.019117 | 6.16E-28 | 2.11E-25 |
|  | ZK813.1 | | 69.63281 | | 3.06073 | 1.61E-15 | 2.10E-13 |
|  | C10G8.4 | | 31.0377 | | 3.066049 | 9.06E-11 | 6.73E-09 |
|  | W02D9.7 | | 59.45009 | | 3.120281 | 1.98E-11 | 1.59E-09 |
|  | F11H8.3 | | 307.9192 | | 3.141754 | 2.02E-15 | 2.60E-13 |
|  | Y22D7AR.10 | | 16.75184 | | 3.187799 | 4.61E-06 | 0.000141 |
|  | W03F11.1 | | 23.64463 | | 3.44672 | 8.94E-10 | 5.87E-08 |
|  | Y45F10C.2 | | 8.540155 | | 3.571755 | 0.000401 | 0.007216 |
|  | K02D7.3 | | 38.57635 | | 3.579531 | 4.33E-16 | 6.12E-14 |
|  | C28D4.13 | | 14.28266 | | 3.742925 | 5.91E-06 | 0.000173 |
|  | W02D9.5 | | 9.850731 | | 3.795467 | 2.46E-05 | 0.000624 |
|  | T15B7.3 | | 52.6874 | | 4.239978 | 6.11E-20 | 1.21E-17 |
