# Supplementary material for: Polyphyletic screen defines distinct classes of plant-derived natural products that oppose tauopathy
Source: Life Sci Alliance. 2025 Nov 17;9(2):e202503393. doi: 10.26508/lsa.202503393 (PMC12623141; doi:10.26508/lsa.202503393)
Supplement: Supplementary file 2 [file LSA-2025-03393_TableS2.docx]

**Table S2. Results and statistical analyses of OFA and OFB treated *C. elegans* in lifespan assay.**

Statistics of lifespan data from Figure 4 and S5.

| Strain | Condition | Maximum survival  (days) | Median survival  ± SE  (days) | % Median survival vs. control | *P* value  (log-rank) treatment vs. control | Combined  Number  (N=3) | Number Censored |
| --- | --- | --- | --- | --- | --- | --- | --- |
| Wild-type  (N2) |  |  |  |  |  |  |  |
| L4 treatment | Control | 24 | 14±0.98 |  |  | 171 | 5 |
|  | OFA 10 μM | 32 | 18±0.61 | 28.57 | < 0.0001 | 120 | 4 |
|  | OFB 10 μM | 32 | 18±0.60 | 28.57 | < 0.0001 | 118 | 12 |
|  | OFAB 10 μM | 29 | 16±0.88 | 14.28 | < 0.0001 | 178 | 15 |
| D5 treatment | Control | 26 | 14±0.1.3 |  |  | 86 | 3 |
|  | OFA 10 μM | 26 | 16±0.56 | 14.28 | < 0.01 | 78 | 5 |
|  | OFB 10 μM | 24 | 14±0.68 | 0 | NS | 82 | 4 |
|  | OFAB 10 μM | 24 | 15±0.95 | 7.14 | < 0.05 | 93 | 2 |
| hTau o/e |  |  |  |  |  |  |  |
| L4 treatment | Control | 22 | 8±0.83 |  |  | 100 | 5 |
|  | OFA 10 μM | 26 | 14±0.39 | 75.00 | < 0.001 | 122 | 8 |
|  | OFB 10 μM | 28 | 14±1.70 | 75.00 | < 0.01 | 129 | 1 |
|  | OFAB 10 μM | 28 | 14±0.43 | 75.00 | < 0.0001 | 107 | 11 |
| D5 treatment | Control | 22 | 9±1.55 |  |  | 109 | 2 |
|  | OFA 10 μM | 24 | 9±0.65 | 0 | NS | 113 | 5 |
|  | OFB 10 μM | 24 | 8±0.74 | 11.11 | NS | 124 | 1 |
|  | OFAB 10 μM | 26 | 12±0.48 | 33.33 | < 0.01 | 104 | 2 |
