# Supplementary material for: Polyphyletic screen defines distinct classes of plant-derived natural products that oppose tauopathy
Source: Life Sci Alliance. 2025 Nov 17;9(2):e202503393. doi: 10.26508/lsa.202503393 (PMC12623141; doi:10.26508/lsa.202503393)
Supplement: Supplementary file 3 [file LSA-2025-03393_TableS3.docx]

**Table S3. Results and statistical analyses of OFA and OFB treated *C. elegans* in progeny assay.**

Statistics of progeny data from Figure 3.

| Strain | Condition | Maximum total brood size  (eggs) | Mean total brood size ± SEM  (eggs) | % mean total brood size vs. control | *P* value  (one-way ANOVA) treatment vs. control | Number |
| --- | --- | --- | --- | --- | --- | --- |
| Wild-type  (N2) | Control | 264 | 249.8±12.59 |  |  | 4 |
| hTau o/e | Control | 59 | 36.56±9.10 |  |  | 6 |
|  | OFA 10 μM | 130 | 87.58±14.14 | 139.55 | < 0.01 | 6 |
|  | OFB 10 μM | 74 | 65.88±8.43 | 80.20 | < 0.05 | 4 |
